# Research Criteria for the Behavioral Variant of Alzheimer Disease: A Systematic Review and Meta-analysis

**DOI:** 10.1001/jamaneurol.2021.4417

**Published:** 2021-12-06

**Authors:** Rik Ossenkoppele, Ellen H. Singleton, Colin Groot, Anke A. Dijkstra, Willem S. Eikelboom, William W. Seeley, Bruce Miller, Robert Jr Laforce, Philip Scheltens, Janne M. Papma, Gil D. Rabinovici, Yolande A. L. Pijnenburg

**Affiliations:** 1 Alzheimer Center Amsterdam, Department of Neurology, Amsterdam Neuroscience, Vrije Universiteit Amsterdam, Amsterdam UMC, Amsterdam, the Netherlands; 2 Lund University, Clinical Memory Research Unit, Lund, Sweden; 3 Department of Pathology, Amsterdam Neuroscience, Amsterdam University Medical Centre, Location VUMC, Amsterdam, the Netherlands; 4 Department of Neurology, Erasmus University Medical Center, Rotterdam, the Netherlands; 5 Memory and Aging Center, Department of Neurology, University of California, San Francisco, San Francisco; 6 Clinique Interdisciplinaire de Mémoire, Centre Hospitalier Universitaire de Québec, Québec, Canada; 7 Department of Radiology and Biomedical Imaging, University of California, San Francisco, San Francisco; 8 Weill Institute for Neurosciences, University of California, San Francisco, San Francisco; 9 Associate Editor, *JAMA Neurology*

## Abstract

**Importance:**

The behavioral variant of Alzheimer disease (bvAD) is characterized by early and predominant behavioral deficits caused by AD pathology. This AD phenotype is insufficiently understood and lacks standardized clinical criteria, limiting reliability and reproducibility of diagnosis and scientific reporting.

**Objective:**

To perform a systematic review and meta-analysis of the bvAD literature and use the outcomes to propose research criteria for this syndrome.

**Data Sources:**

A systematic literature search in PubMed/MEDLINE and Web of Science databases (from inception through April 7, 2021) was performed in duplicate.

**Study Selection:**

Studies reporting on behavioral, neuropsychological, or neuroimaging features in bvAD and, when available, providing comparisons with typical amnestic-predominant AD (tAD) or behavioral variant frontotemporal dementia (bvFTD).

**Data Extraction and Synthesis:**

This analysis involved random-effects meta-analyses on group-level study results of clinical data and systematic review of the neuroimaging literature. The study was performed following Preferred Reporting Items for Systematic Reviews and Meta-analyses (PRISMA) guidelines.

**Main Outcomes and Measures:**

Behavioral symptoms (neuropsychiatric symptoms and bvFTD core clinical criteria), cognitive function (global cognition, episodic memory, and executive functioning), and neuroimaging features (structural magnetic resonance imaging, [^18^F]fluorodeoxyglucose-positron emission tomography, perfusion single-photon emission computed tomography, amyloid positron emission tomography, and tau positron emission tomography).

**Results:**

The search led to the assessment of 83 studies, including 13 suitable for meta-analysis. Data were collected for 591 patients with bvAD. There was moderate to substantial heterogeneity and moderate risk of bias across studies. Cases with bvAD showed more severe behavioral symptoms than tAD (standardized mean difference [SMD], 1.16 [95% CI, 0.74-1.59]; *P* < .001) and a trend toward less severe behavioral symptoms compared with bvFTD (SMD, −0.22 [95% CI, −0.47 to 0.04]; *P* = .10). Meta-analyses of cognitive data indicated worse executive performance in bvAD vs tAD (SMD, −1.03 [95% CI, −1.74 to −0.32]; *P* = .008) but not compared with bvFTD (SMD, −0.61 [95% CI, −1.75 to 0.53]; *P* = .29). Cases with bvAD showed a nonsignificant difference of worse memory performance compared with bvFTD (SMD, −1.31 [95% CI, −2.75 to 0.14]; *P* = .08) but did not differ from tAD (SMD, 0.43 [95% CI, −0.46 to 1.33]; *P* = .34). The neuroimaging literature revealed 2 distinct bvAD neuroimaging phenotypes: an AD-like pattern with relative frontal sparing and a relatively more bvFTD-like pattern characterized by additional anterior involvement, with the AD-like pattern being more prevalent.

**Conclusions and Relevance:**

These data indicate that bvAD is clinically most similar to bvFTD, while it shares most pathophysiological features with tAD. Based on these insights, we propose research criteria for bvAD aimed at improving the consistency and reliability of future research and aiding the clinical assessment of this AD phenotype.

## Key Points

**Question:**

How is the behavioral variant of Alzheimer disease (bvAD) associated with typical AD (tAD) and behavioral variant frontotemporal dementia (bvFTD) in terms of clinical presentation and neuroimaging signatures?

**Findings:**

This systematic review and meta-analysis found that, at time of diagnosis, bvAD showed more severe neuropsychiatric symptoms and other behavioral deficits compared with tAD. Two distinct neuroimaging phenotypes were observed across reported bvAD cases: an AD-like pattern with relative frontal sparing and a relatively more bvFTD-like pattern with both posterior and anterior involvement, with the AD-like bvAD neuroimaging phenotype being the most prevalent.

**Meaning:**

This analysis found that bvAD is clinically most reminiscent of bvFTD, while it shares most pathophysiological features with tAD; the research criteria are aimed at improving the consistency and reliability of future research and potentially aiding in the clinical assessment of bvAD.

## Teaser

This systematic review and meta-analysis reviews literature on the behavioral variant of Alzheimer disease and uses the outcomes to propose research criteria for this syndrome.

## Introduction

Alzheimer disease (AD) is a heterogenous disease that can manifest with both amnestic and nonamnestic clinical presentations.^1^ Several atypical (ie, non–memory predominant) variants of AD have been described, including posterior cortical atrophy, logopenic variant primary progressive aphasia, corticobasal syndrome due to AD, and dysexecutive AD.^2^ The behavioral variant of Alzheimer disease (bvAD) represents another, rare variant of AD that is characterized by early and predominant behavioral deficits and personality changes caused by AD pathology. The bvAD clinical syndrome overlaps substantially with that of the behavioral variant of frontotemporal dementia (bvFTD) and approximately 10% to 40% of clinically diagnosed bvFTD cases have positive AD biomarkers and/or neuropathologically confirmed AD.^3,4,5,6^ This highlights a major diagnostic challenge, which is even more pertinent with the recent accelerated approval of aducanumab by the US Food and Drug Administration to reduce cerebral amyloid-β in early symptomatic AD.^7^ Although bvAD is acknowledged as a clinical entity in recent diagnostic and research criteria for AD dementia,^8,9^ currently no criteria exist that provide specific recommendations for the diagnosis of bvAD. This is in contrast with other AD variants^10,11,12^ and limits reliable and reproducible classification of bvAD as well as uniform scientific reporting.

The current literature on bvAD includes relatively few studies with typically small sample sizes that have reported several inconsistent findings. To better understand the bvAD phenotype, we performed a systematic review and meta-analysis of the clinical, neuroimaging, and neuropathology bvAD literature and applied the outcomes to develop research criteria for bvAD. With this work, we aim to improve the consistency and reliability of future research and potentially aid in the clinical assessment of bvAD.

## Methods

### Search Strategy, Selection, and Outcomes

This study was conducted following prespecified methods (PROSPERO registration number: CRD42021243497) and reported following the Preferred Reporting Items for Systematic Reviews and Meta-analyses (PRISMA) reporting guidelines. We performed a systematic literature search in PubMed/MEDLINE and Web of Science databases. We searched studies including clinically diagnosed (1) AD cases with so-called frontal or behavioral presentations or (2) bvFTD cases with neuropathological evidence of AD (full database queries are in eTable 1 in the Supplement). We included peer-reviewed articles, written in English, and presenting original research with human data only. Screening was first conducted at the title or abstract level in Rayyan.^13^ Reference lists were additionally cross-checked for eligible studies. Two independent reviewers (R.O. and E.H.S.) screened titles and abstracts. Ambiguous records were discussed with a third author (Y.A.L.P.) to reach consensus. Studies were eligible when (1) they included cases or groups of patients presenting with early and predominant behavioral changes with a clinical diagnosis, biomarker support, and/or neuropathological evidence of AD and (2) behavioral/neuropsychiatric, neuropsychological, neuroimaging, and/or neuropathological data were presented. Studies were excluded when (1) they described patients with isolated executive dysfunction in the absence of behavioral symptoms and (2) there was biomarker and/or neuropathological evidence for a non-AD pathology as the primary pathology. Studies were only eligible for the meta-analysis if a bvAD group was compared with typical AD (tAD) and/or bvFTD groups. We extracted demographic (age and sex), clinical (behavioral features per bvFTD criteria^14^ or neuropsychiatric symptoms per Neuropsychiatric Inventory [NPI^15^]), neuropsychological (Mini-Mental State Examination [MMSE] and memory and executive function tests), neuroimaging (structural magnetic resonance imaging [MRI], [^18^F]fluorodeoxyglucose [FDG]–positron emission tomography [PET], perfusion single-photon emission computed tomography, amyloid-PET, and tau-PET), and neuropathological (amyloid-β and tau) characteristics from all studies. After eligibility assessment for inclusion, meta-analyses were constructed using pooled clinical data (behavioral or neuropsychiatric questionnaires), neuropsychological data (MMSE and memory and executive functioning tests), and neuropathological data (amyloid-β and tau load in medial temporal lobe, occipital cortex, and frontal regions; eTable 2 in the Supplement). The lack of uniform reporting of effect sizes among neuroimaging methods across studies did not allow a meta-analysis; hence these findings were analyzed using systematic review (Table).

**Table.** Neuroimaging Studies in the Behavioral Variant of AD

Source	Participants	Age, mean (SD), y	Male, %	MMSE score, mean (SD)	AD confirmation methods	Contrasts	Findings
**Magnetic resonance imaging**							
Ossenkoppele et al,^16^ 2015	55 With bvAD	64.7 (8.8)	72.7	22.5 (5.4)	CSF, PET, or autopsy	58 With tAD, 59 with bvFTD, and 61 with CU	Predominant temporoparietal pattern and no differences between bvAD and tAD
Perry et al,^17^ 2017^a^	15 With bvAD	62.8 (43-83)^b^	66.7	NA	Autopsy	98 With bvFTD	Moderate atrophy in frontoinsula and anterior cingulate regions
Phillips et al,^18^ 2018	22 With b/dAD	64.3 (8.2)	50.0	19.6 (8.4)	CSF or autopsy	22 With tAD and with 115 CU	Staging scheme based on cross-sectional magnetic resonance imaging data indicated early frontotemporal and insular atrophy and spread toward frontotemporal and parietal cortex
Phillips et al,^19^ 2019^c^	12 With bvAD	16.0 (13.5-18.0)^d^	58.3	23.0 (17.0-26.0)	CSF or autopsy	17 With tAD	Anterior insula, frontotemporal, angular gyrus, and middle temporal atrophy
Singleton et al,^20^ 2020^a^	29 With bvAD	64.4 (9.4)	59.0	22.0 (5.9)	CSF, PET, or autopsy	28 With tAD, 28 with bvFTD, and 34 with CU	Larger amygdala gray matter volume in bvAD vs tAD
Therriault et al,^21^ 2020	15 With b/dAD	65.9 (8.8)	40.0	19.6 (5.3)	Amyloid- and tau-PET	25 With tAD and 131 with CU	Lateral and medial parietal and prefrontal atrophy in bvAD; no differences between bvAD vs tAD
**Fluorodeoxyglucose-PET or hypoperfusion single-photon emission computed tomography**							
Snowden et al,^22^ 2007	12 With bvAD	49 (8)	75.0	NA	NA	321 With tAD	Hypoperfusion extending into frontal regions occurred more commonly in patients with so-called frontal behavioral features
Woodward et al,^23^ 2015	13 With bvAD	81.6 (4.1)	61.5	23.9	NA	40 With tAD	Greater frontotemporal and parietal hypometabolism in bvAD vs tAD
Wang et al,^24^ 2019	13 With b/dAD	68.0 (3.4)	30.8	17.0 (5.6)	Amyloid-PET	38 With tAD and 20 with CU	Dorsolateral hypometabolism in the medial prefrontal and dorsolateral frontal cortex
Bergeron et al,^25^ 2020	8 With b/dAD	61.6	NA	20.7	CSF or PET	40 With atypical AD and 12 with FTD	Most hypometabolic regions of interest in b/dAD were middle temporal gyrus, posterior cingulate, and angular gyrus
Singleton et al,^20^ 2020^a^	19 With bvAD	66.1 (7.4)	58.0	21.8 (5.7)	CSF, PET, or autopsy	18 With tAD, 18 with bvFTD, and 31 with CU	No hypometabolic differences in direct contrasts but relatively greater frontoinsular involvement in bvAD vs tAD in contrast against cases with CU
Sala et al,^26^ 2020	15 With b/dAD	62.5 (5.7)	66.7	16.5 (5.2)	CSF	22 With tAD	Temporoparietal, dorsolateral, and orbitofrontal hypometabolism, with greatest (>90%) hypometabolism in the middle and superior frontal gyrus
Bergeron et al,^27^ 2020	6 With b/dAD	59.5 (7.9)	75.0	22.3 (5.9)	CSF or PET	8 With bvFTD and 10 with tAD	Two of 6 showed a predominantly frontal and temporoparietal pattern, 2 showed a mild frontal pattern, and the final 2 showed a temporoparietal-predominant pattern
Lehingue et al,^28^ 2021	20 With bvAD	71.5 (66-76)^d^	65.0	25 (21-26)	CSF	22 With tAD and 36 with bvFTD	No significant differences between bvAD vs tAD or bvFTD
**Amyloid PET**							
Wang et al,^24^ 2019	13 With b/dAD	68.0 (3.4)	30.8	17.0 (5.6)	Amyloid-PET	38 With tAD and 20 with CU	No differences among AD groups
Therriault et al,^21^ 2020	15 With b/dAD	65.9 (8.8)	40.0	19.6 (5.3)	Amyloid- and tau-PET	25 With tAD and 131 with CU	No differences among AD groups
**Tau PET**							
Therriault et al,^21^ 2020	15 With b/dAD	65.9 (8.8)	40.0	19.6 (5.3)	Amyloid- and tau-PET	25 With tAD and 131 with CU	b/dAD vs tAD: elevated uptake in the anterior cingulate, medial prefrontal, and frontal insula cortices in b/dAD vs tAD
Singleton et al,^29^ 2021	7 With bvAD	69.1 (8.4)	85.7	21.7 (2.8)	CSF, PET, or autopsy	205 With tAD	Three of 7 prominent lateral frontal and temporoparietal uptake, 1 with medial prefrontal uptake, 2 with lateral temporal uptake, and 1 with temporoparietal uptake

Abbreviations: AD, Alzheimer disease; b/dAD, behavioral/dysexecutive Alzheimer disease; bvAD, behavioral variant of Alzheimer disease; bvFTD, behavioral variant of frontotemporal dementia; CU, cognitively unimpaired; CSF, cerebrospinal fluid; MMSE, Mini-Mental State Examination; NA, not applicable; PET, positron emission tomography; tAD, typical Alzheimer disease.

^a^ Cases partially overlap with Ossenkoppele et al.^16^

^b^ Reported as mean (range).

^c^ Cases partially overlap with Phillips et al.^18^

^d^ Reported as median (IQR).

### Statistical Analysis

Meta-analysis was used to examine whether bvAD differed from tAD and bvFTD in terms of behavioral/neuropsychiatric and neuropsychological features and bvAD differed from tAD in the distribution of amyloid-β and tau pathology defined at autopsy. Missing data were requested from the authors of 3 studies (and all 3 responded).^18,27,28^ We calculated the pooled standardized mean differences and 95% CIs using Hedges *g* random-effects models in the “meta” package of R version 4.0.2 (R Foundation for Statistical Computing), with a significance level of *P* < .05. We used random effects because we assumed that the true effect size would be study dependent because of high heterogeneity in samples, methods, and outcomes among studies.

Statistical heterogeneity for the meta-analyses was assessed using the *I*^2^ statistic, with *I*^2^ greater than 75% indicating substantial heterogeneity. Heterogeneity across studies was substantial for analyses including behavioral and neuropsychiatric symptoms, memory and executive measures (*I*^2^ range, 70%-96%), and moderate for analyses including neuropathological data (*I*^2^ range, 0%-51%). Publication bias was assessed by visual inspection of funnel plots, which indicated substantial publication bias (eFigures 1-3 in the Supplement). Two authors (E.H.S. and C.G.) independently assessed risk of bias using the Risk of Bias in Nonrandomized Studies of Interventions (ROBINS-I) risk of bias tool for nonrandomized studies. The overall risk of bias was serious for 2 studies and moderate for 11 studies in the meta-analysis (eTable 3 and eFigure 4 in the Supplement).

We additionally calculated the prevalence of each behavioral feature in the core clinical bvFTD criteria^14^ (ie, presence of disinhibition, apathy, lack of empathy, compulsiveness, and hyperorality) and the 12 items of the neuropsychiatric inventory (NPI and prevalence score ≥1) across studies and compared bvAD, bvFTD, and tAD groups using χ^2^ tests. For the NPI analysis only, we examined 769 amyloid-β–positive patients with tAD from the Amsterdam Dementia Cohort.^30^

## Results

### Participants

The systematic literature search yielded 1257 records, of which 116 studies were assessed at full-text level for eligibility and 83 studies^5,16,17,18,19,20,21,22,23,24,26,27,28,31,32,33,34,35,36,37,38,39,40,41,42,43,44,45,46,47,48,49,50,51,52,53,54,55,56,57,58,59,60,61,62,63,64,65,66,67,68,69,70,71,72,73,74,75,76,77,78,79,80,81,82,83,84,85,86,87,88,89,90,91,92,93,94,95,96,97^ met inclusion criteria (eFigure 5 in the Supplement for flowchart). Confirmation of AD pathology was present in 91.1% of cases with bvAD based on autopsy data (36 studies^5,16,17,18,19,20,22,29,31,33,34,35,39,40,42,44,49,51,52,54,55,56,57,59,62,66,69,70,76,77,79,81,82,87,90,96^; n = 334), genetic data (9 studies^47,50,60,63,65,67,68,80,86^; n = 21), or biomarker data (31 studies^3,18,19,20,21,24,25,26,27,28,29,32,38,63,70,71,72,73,74,75,77,83,84,85,88,89,91,92,94,95,97^; n = 262), while no information was available in 9.9% of cases (11 studies^22,23,36,37,41,43,45,46,53,64,78^ including 68 cases of bvAD). Thirteen studies were eligible for meta-analysis. eTable 4 in the Supplement provides an overview of the participant characteristics for all 83 included studies. Across these studies, 591 patients with bvAD were enrolled, with a mean (SD) age at diagnosis of 62.0 (7.3) years, and 226 participants (38.2%) were women. The mean (SD) MMSE score was 20.1 (5.9), and 281 participants (47.5%) carried an *APOE*ε4 allele.

### Behavioral/Neuropsychiatric Symptoms

**Figure 1.**
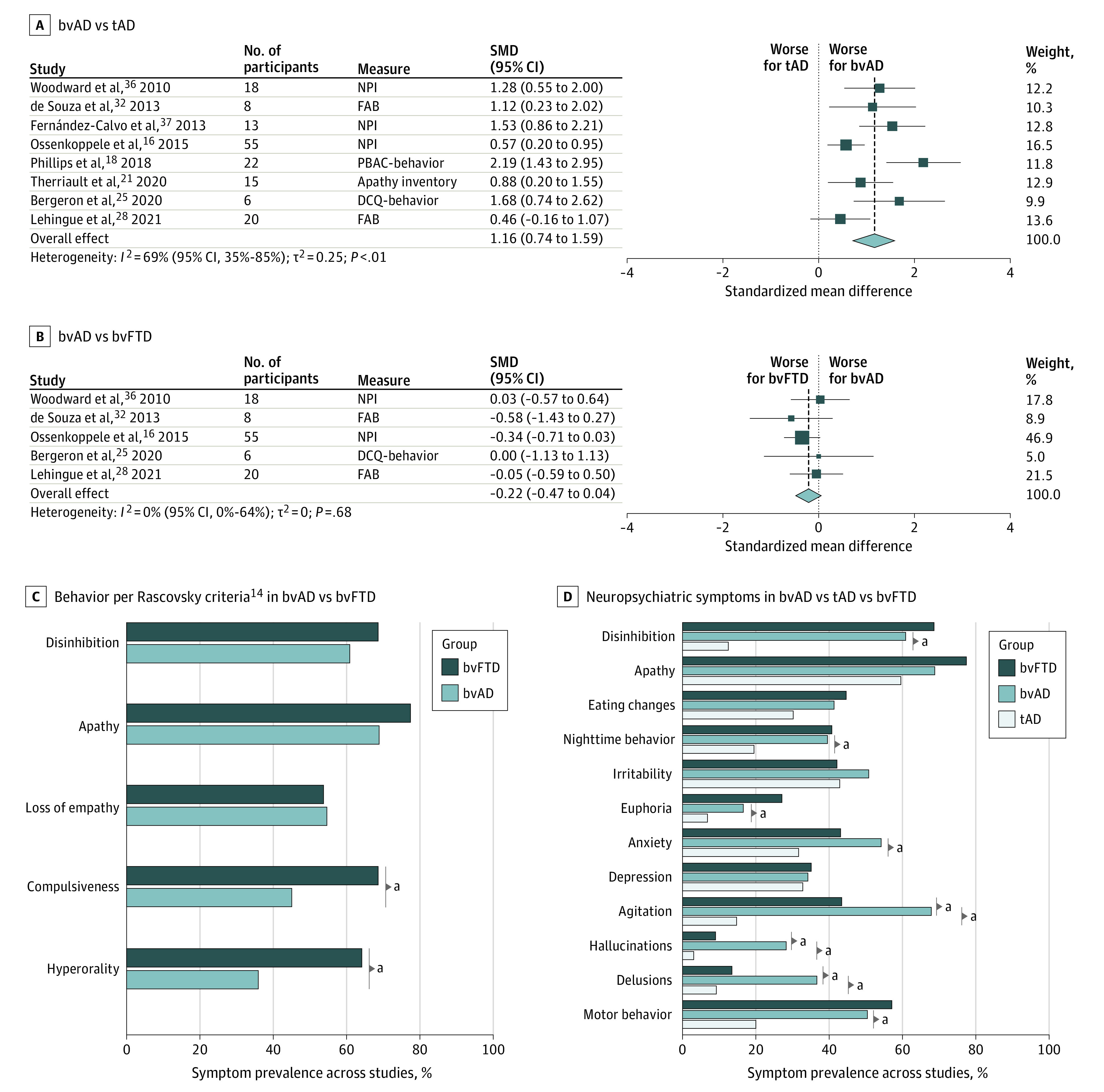
Meta-analyses of Behavioral and Neuropsychiatric Features Results of meta-analyses on behavioral and neuropsychiatric total scores between the behavioral variant of Alzheimer disease (bvAD) and typical Alzheimer disease (tAD) (A) and bvAD and the behavioral variant of frontotemporal dementia (bvFTD) (B). Mean weighted percentages of participants per diagnostic group fulfilling specific bvFTD core clinical features proposed by Rascovsky et al^14^ (C) or presence of specific neuropsychiatric symptoms measured using the Neuropsychiatric Inventory (NPI) (D). DCQ indicates Dépistage Cognitif de Québec; FAB, Frontal Assessment Battery; PBAC, Philadelphia Brief Assessment of Cognition; SMD, standardized mean difference. ^a^*P* < .05.

Meta-analysis indicated that patients with bvAD showed more severe behavioral and neuropsychiatric symptoms than patients with tAD (standardized mean difference [SMD], 1.16 [95% CI, 0.74-1.59]; *P* < .001) and a nonsignificant difference in severe behavioral/neuropsychiatric symptoms compared with bvFTD (SMD, −0.22 [95% CI, −0.47 to 0.04]; *P* = .10; Figure 1A). Results remained similar when separating bvFTD core criteria and neuropsychiatric features (eFigure 6 in the Supplement).

Next, we compared proportions of bvFTD features and NPI items as reported in previous studies (Figure 1B; eTable 5 in the Supplement^16,17,19,31,32,33,34^). We used amyloid-β–positive patients with tAD from the Amsterdam Dementia Cohort (mean [SD] age, 65.9 [7.7] years; 403 women [52.4%]; mean [SD] MMSE score, 20.3 [5.1]). Compared with bvFTD, patients with bvAD less frequently showed compulsive behaviors (45.0% vs 68.5%; χ^2^ = 22.5; *P* < .001) and hyperorality (35.9% vs 64.1%; χ^2^ = 32.8; *P* < .001) but showed no differences on disinhibition (60.8% vs 68.6%; χ^2^ = 2.8; *P* = .10), apathy (68.8% vs 77.4%; χ^2^ = 3.7; *P* = .05), and lack of empathy (54.6% vs 53.6%; χ^2^ = 0.1; *P* = .83). On the NPI, patients with bvAD more frequently showed agitation (67.9% vs 43.4%; χ^2^ = 8.8), hallucinations (28.2% vs 9.0%; χ^2^ = 12.8), and delusions (36.6% vs 13.4%; χ^2^ = 13.4) compared with bvFTD (*P* < .001). Furthermore, those with bvAD more frequently showed nighttime behaviors (39.6% vs 19.5%; χ^2^ = 12.9), euphoria (16.6% vs 6.8%; χ^2^ = 7.9), anxiety (54.2% vs 31.7%; χ^2^ = 10.8), agitation (67.9% vs 14.8%; χ^2^ = 90.3), hallucinations (28.2% vs 3.1%; χ^2^ = 71.2), delusions (36.6% vs 9.2%; χ^2^ = 37.2), and motor behaviors (50.4% vs 19.8%; χ^2^ = 26.2) compared with patients with tAD (*P* < .01).

### Cognition

**Figure 2.**
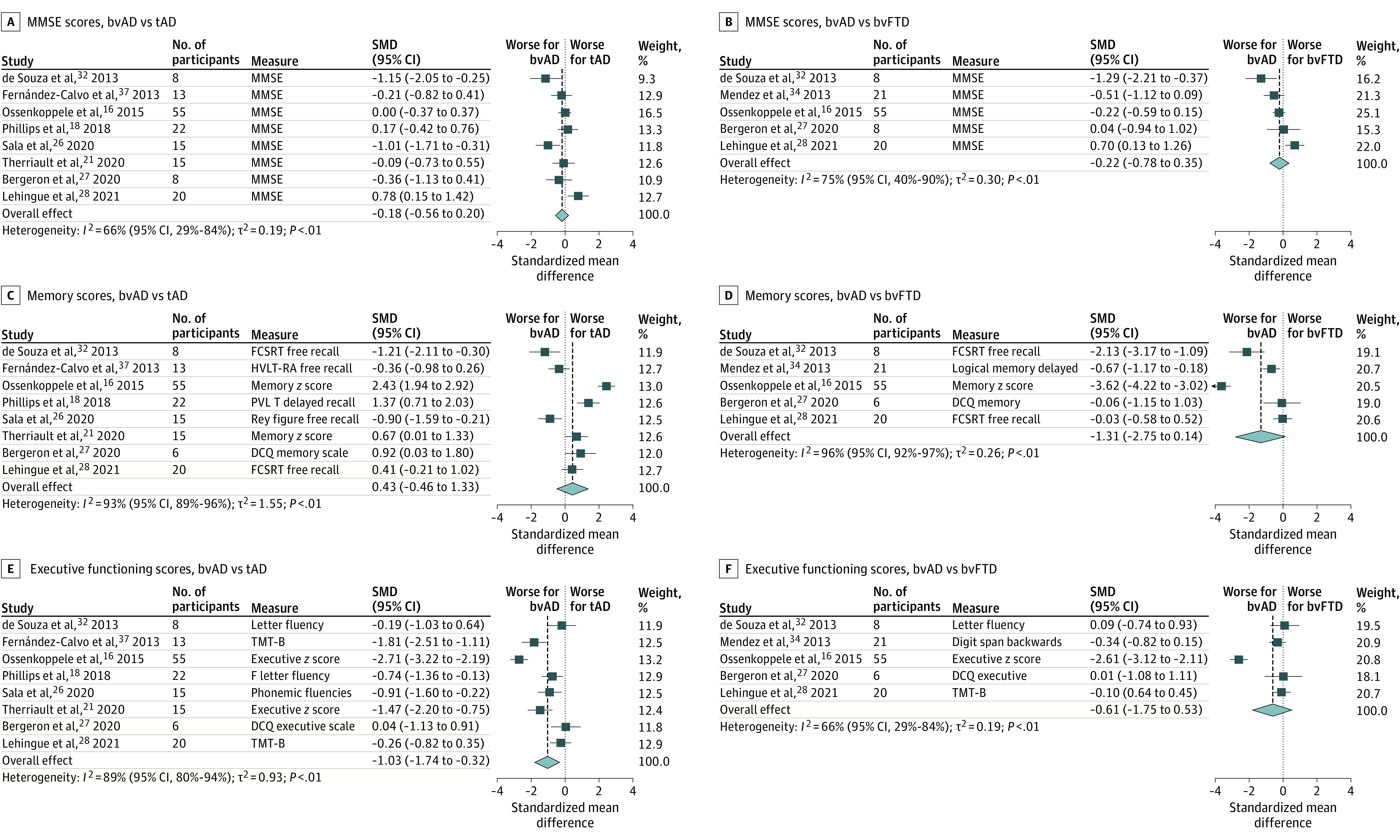
Meta-analyses of Cognitive Performance Results of meta-analyses on Mini-Mental State Examination (MMSE; A and B), episodic memory (C and D), and executive function (E and F) for the contrast of behavioral variant of Alzheimer disease (bvAD) vs typical Alzheimer disease (tAD) and the behavioral variation of frontotemporal dementia (bvFTD). SMD indicates standardized mean difference.

Meta-analyses of cognitive data indicated that at initial assessment bvAD, patients showed no differences on MMSE compared with tAD (SMD, −0.18 [95% CI, −0.56 to 0.20]; *P* = .35) and bvFTD (SMD, −0.22 [95% CI, −0.78 to 0.35]; *P* = .46; Figure 2). Patients with bvAD showed worse executive performance compared with tAD (SMD, −1.03 [95% CI, −1.74 to −0.32]; *P* = .008) but not compared with bvFTD (SMD, −0.61 [95% CI, −1.75 to 0.53]; *P* = .29). Finally, bvAD showed a trend toward worse memory performance compared with bvFTD (SMD, −1.31 [95% CI, −2.75 to 0.14]; *P* = .08) but did not differ from tAD (SMD, 0.43 [95% CI, −0.46 to 1.33]; *P* = .34).

### Neuroimaging

The Table provides an overview of neuroimaging studies in bvAD. Structural MRI studies (16 studies; 92 participants) showed temporoparietal-predominant,^16^ frontotemporal-predominant and insular-predominant,^17,18,19^ or frontoparietal-predominant^21^ atrophy patterns across patients with bvAD. Cases of bvAD did not differ from tAD in 3 studies^16,20,21^ and showed moderately more involvement of frontal regions in bvAD compared with tAD in 3 other studies.^17,18,19^ Studies assessing glucose metabolism with [^18^F]FDG-PET or perfusion with single-photon emission computed tomography (7 studies; 88 participants) also showed heterogeneous results, ranging from a predominantly temporoparietal hypometabolic pattern^20,25^ to a mixed frontal and temporoparietal^23,26,27,28^ or predominantly frontal pattern.^24^ Amyloid-PET studies (2 studies^21,24^; 28 participants) showed no differences in amyloid-β burden or distribution between patients with bvAD vs tAD. For tau-PET (2 studies; 22 participants), 1 study^21^ showed a temporoparietal pattern with higher uptake in anterior regions in bvAD compared with tAD, whereas another^29^ study showed heterogeneous patterns across patients with bvAD. Findings on functional connectivity (3 studies^19,20,24^; 54 participants) and white matter hyperintensities (1 study^20^; 29 participants) in bvAD are presented in eTable 6 in the Supplement.

**Figure 3.**
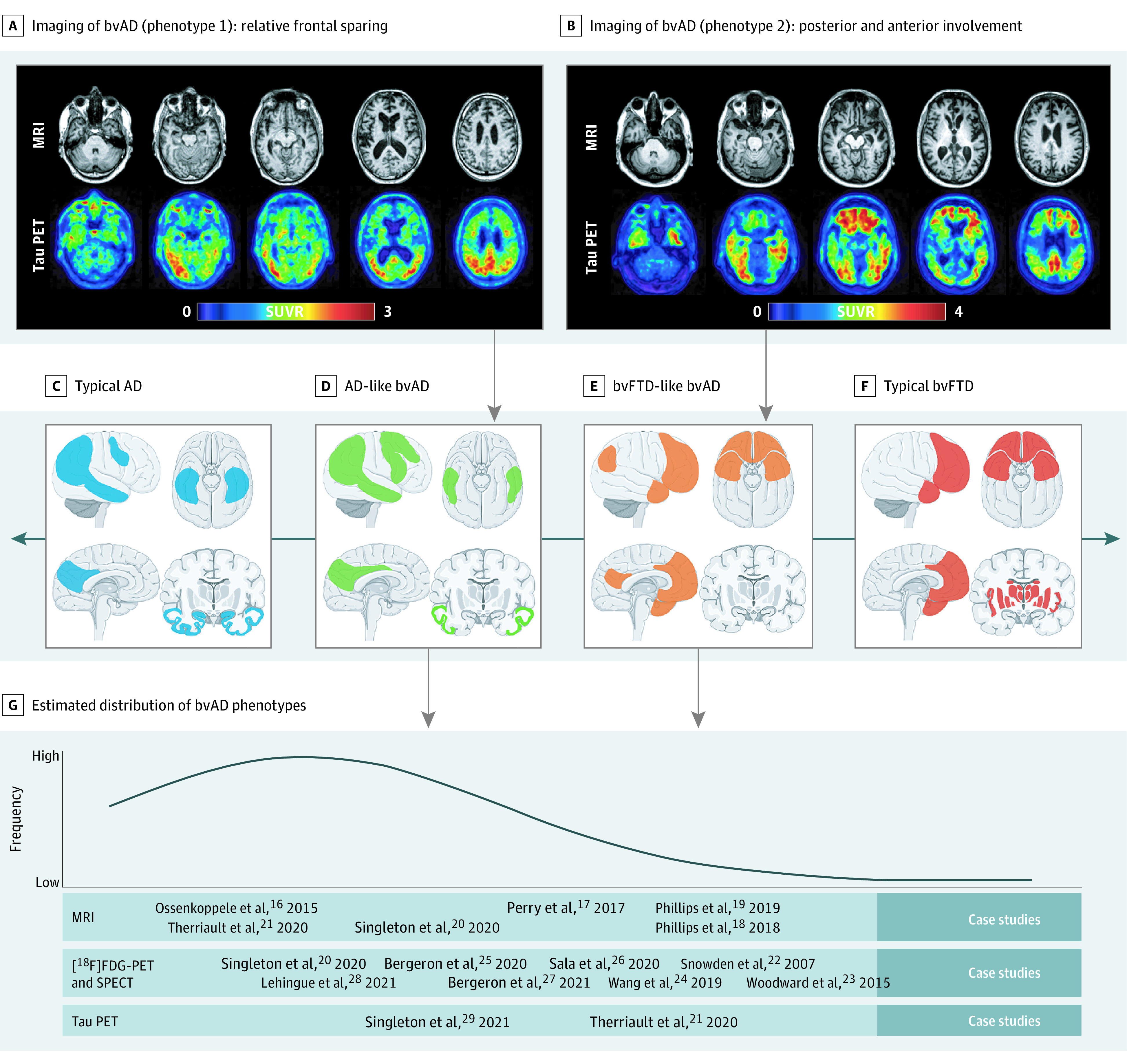
Neuroimaging Features in the Behavioral Variant of Alzheimer Disease (bvAD) A and B, Two cases that serve as examples of 2 distinct bvAD neuroimaging phenotypes: an Alzheimer disease–like atrophy and tau load pattern with relative frontal sparing and a more behavioral variant of frontotemporal dementia (bvFTD)–like atrophy and tau load pattern with both posterior and anterior involvement. The tau positron emission tomography (PET) scans were performed using [^18^F]flortaucipir, and magnetic resonance imaging (MRI) was conducted on a 3-T scanner. C-F, Proposed neuroimaging phenotypes as part of a spectrum that ranges from a typical Alzheimer disease regional distribution to a classical bvFTD regional distribution. The brain template images were obtained from https://smart.servier.com/. G, Literature-informed estimated distribution of the regional distribution in bvAD, indicating that typical AD and bvAD-AD–like patterns are more common than bvAD-bvFTD–like and typical bvFTD. FDG indicates fluorodeoxyglucose; SPECT, single-photon emission computed tomography; SUVR, standardized uptake value ratio.

We distilled 2 distinct bvAD neuroimaging phenotypes from the literature, characterized by either relative frontal sparing (more AD-like) or by both posterior and anterior involvement (more bvFTD-like) (Figure 3A). We propose that these phenotypes occur on a continuum (Figure 3B), with the more AD-like phenotype being most prevalent (Figure 3C).

### Neuropathology

In line with amyloid and tau PET findings, the meta-analyses on neuropathological data^18,29,31,35^ showed that bvAD and tAD did not differ in the neuropathological burden of amyloid-β (3 studies^18,31,35^; 20 participants) across frontal regions (SMD, 0.23 [95% CI, −0.36 to 0.81]; *P* = .45), medial temporal lobe (SMD, −0.06 [95% CI, −0.65 to 0.53]; *P* = .84), or occipital cortex (SMD, −0.16 [95% CI, −1.05 to 0.73]; *P* = .73; eFigure 7 in the Supplement). Furthermore, there was no difference in tau burden (4 studies^18,29,31,35^; 28 participants) across frontal regions (SMD, −0.05 [95% CI, −0.56 to 0.46]; *P* = .84), medial temporal lobe (SMD, 0.32 [95% CI, −0.19 to 0.83]; *P* = .22), or occipital cortex (SMD, −0.36 [95% CI, −0.95 to 0.23]; *P* = .24; eFigure 7 in the Supplement).

## Discussion

In this systematic review and meta-analysis, we found that bvAD is clinically most reminiscent of bvFTD while it shares most pathophysiological features with tAD. Based on these insights, we provide research criteria for bvAD aimed at improving the consistency and reliability of future research and aiding in future clinical assessments.

### Systematic Review and Meta-analyses

bvAD phenotype typically presents at a young age (mean [SD] age, 62.0 [7.3] years at diagnosis), is more frequently found in men than women (61.7% vs 38.2%, in line with bvFTD but in contrast with tAD^98^), and has a lower frequency of *APOE*ε4 carriership compared with tAD (47.5% vs 66.1%^99^). Clinically, bvAD shows a milder behavioral profile compared with bvFTD, with less compulsivity and hyperorality but greater prevalence of neuropsychiatric symptoms, such as agitation, delusions, and hallucinations. By definition, bvAD shows greater impairment on a range of behavioral and neuropsychiatric measures compared with tAD. The directionality of findings in the meta-analyses of cognitive data suggest that bvAD might show greater memory and executive function deficits compared with bvFTD and relatively better memory function and worse executive functioning compared with tAD, but further research in larger cohorts is needed to confirm the significance of these findings. The neuroimaging methods were too heterogenous across studies to conduct a formal meta-analysis, but a systematic review revealed 2 distinct phenotypes of brain atrophy, hypometabolism, and tau pathology in bvAD, with many cases likely occurring on a continuum. The most prevalent bvAD neuroimaging phenotype is an AD-like pattern involving bilateral temporoparietal regions with limited involvement of the frontal cortex. This observation is congruent with our meta-analysis on neuropathological data showing that patients with bvAD were indistinguishable from patients with tAD in both amyloid-β and tau load and spatial distribution. The other bvAD phenotype is characterized by a more bvFTD-like neuroimaging pattern, including posterior and anterior regions (eg, anterior cingulate cortex, frontal insula, temporal poles) located in brain networks (eg, the salience network) that are engaged during socioemotional processing of information.^100^ Altogether, our systematic review and meta-analyses further refine the bvAD phenotype but also highlight the need for larger studies with more uniform methods and inclusion and exclusion criteria.

### Research Criteria for bvAD

Our main objective was to propose research criteria for bvAD guided by the results of the systematic review and meta-analyses. The criteria are based on consensus between all authors, including neurologists, neuropsychologists, neuropathologists, and neuroscientists. To facilitate widespread use but also take into account the complexity of this phenotype, we offer 4 levels of evidence (Box). The first level (clinical bvAD) can be established solely based on clinical information, while the second and third levels (possible bvAD and probable bvAD) add biomarker confirmation of amyloid-β and tau pathology. The fourth level (definite bvAD) is assigned through histopathological or genetic confirmation of AD (ie, by the presence of pathogenic *APP, PSEN1*, or *PSEN2* genetic variations) in conjunction with a bvAD clinical syndrome.

Box. **Research Criteria for the Behavioral Variant of Alzheimer Disease (bvAD)**

**Clinical bvAD**

1. The clinical syndrome is characterized by:
  - Early, persistent, predominant, and progressive change or exacerbation of at least 2 of 5 core behavioral features of the diagnostic criteria for behavioral variant frontotemporal dementia (Rascovsky et al^14^):
    - Behavioral disinhibition (1 of the following symptoms must be present):
      - Socially inappropriate behavior
      - Loss of manners or decorum
      - Impulsive, rash, or careless actions
    - Apathy or inertia (1 of the following symptoms must be present):
      - Apathy
      - Inertia
    - Loss of empathy or sympathy (1 of the following symptoms must be present):
      - Diminished response to other people’s needs and feelings
      - Diminished social interest, interrelatedness, or personal warmth
    - Perseverative, stereotyped, or compulsive or ritualistic behavior (1 of the following symptoms must be present):
      - Simple, repetitive movements
      - Complex, compulsive, or ritualistic behaviors
      - Stereotypy of speech
    - Hyperorality and dietary changes (1 of the following symptoms must be present):
      - Altered food preferences
      - Binge eating or increased consumption of alcohol or cigarettes
      - Oral exploration or consumption of inedible objects
  - In addition, documented impairment in executive functions and/or episodic memory with relatively preserved language and visuospatial abilities.
2. Criteria for clinical bvAD are not met if the behavioral deficits are (better) accounted for by another concurrent (active) neurological (eg, Lewy body dementia) or nonneurological medical (eg, psychiatric) comorbidity, a known genetic variant associated with familial behavioral variant of frontotemporal dementia, or the use of medication.
3. Supportive features (not mandatory; categories A and B must be met):
  - Presence of hallucinations and/or delusions.
  - Alzheimer disease–specific (ie, temporoparietal pattern) and/or behavioral variant of frontotemporal dementia–specific neuroimaging features (ie, frontotemporal pattern) on magnetic resonance imaging, computed tomography, perfusion single-photo emission computed tomography, and/or fluorodeoxyglucose–positron emission tomography.

**Possible bvAD**

1. Meets criteria for clinical bvAD and
2. There is in vivo biomarker evidence for the presence of (1) β-amyloid pathology on amyloid positron emission tomography and/or in cerebrospinal fluid and/or (2) tau pathology in cerebrospinal fluid and/or plasma.

**Probable bvAD**

1. Meets criteria for clinical bvAD or possible bvAD, with additional in vivo tau positron emission tomography evidence for the presence of neocortical tau aggregates.

**Definite bvAD**

1. Meets criteria for clinical bvAD, possible bvAD, or probable bvAD, and
2. Presence of AD is established by
  - Histopathological indication of AD as the primary pathology on biopsy or at autopsy, or
  - Presence of a known genetic variant associated with familial AD.

Several issues warrant further explanation. First, both the literature and our clinical experience align with the notion that bvAD is a combined cognitive and behavioral clinical syndrome. We previously showed that cognitive impairment was among the first symptoms reported by patients and caregivers in approximately 75% of bvAD cases.^16^ In addition, our meta-analysis suggests that episodic memory performance in bvAD is intermediate between tAD and bvFTD, while bvAD shows greater executive dysfunction compared with bvFTD (Figure 2). To enhance the discriminative accuracy between bvAD and bvFTD, objectively confirmed impairment in either memory or executive domains is therefore mandatory. To achieve this, we recommend a full neuropsychological evaluation rather than use of relatively crude dementia screening tests. In addition, 2 of 5 behavioral features of the diagnostic criteria for bvFTD^14^ (ie, disinhibition, apathy, lack of empathy, compulsiveness, and hyperorality) must be present. Note that the sixth bvFTD criterion (ie, a dysexecutive neuropsychological profile) was removed because documented memory and/or executive function deficits are required for a bvAD diagnosis. The 2-of-5 criterion was selected to sufficiently distinguish bvAD from tAD but also acknowledge the generally milder behavioral profile in bvAD compared with bvFTD (in which 3 of 6 bvFTD criteria must be present). Second, despite clinically significant differences between bvAD and both bvFTD and tAD (Figure 1), we deemed it premature to include hallucinations and delusions in the core research criteria because these observations were derived from only 2 studies.^33,34^ Instead, they were added as supportive features, and future prospective studies are needed to assess whether they should be incorporated in the core criteria for bvAD. Third, most AD variants have a clear neurodegenerative signature on MRI and/or [^18^F]FDG-PET that corresponds with their clinical phenotype, such as left-hemispheric predominance in logopenic variant primary progressive aphasia or occipitotemporal or occipitoparietal damage in posterior cortical atrophy.^10,11^ However, the neuroimaging literature in bvAD is highly inconsistent. Some studies (mainly case studies or case series) showed anterior neurodegenerative patterns that resemble bvFTD, but most group studies showed either a mix of anterior and posterior involvement or a posterior-predominant pattern.^16,17,18,19,20,21,22,25,36,37^ Contrary to posterior cortical atrophy and logopenic variant primary progressive aphasia, we therefore did not incorporate MRI, computed tomography, single-photon emission computed tomography, or [^18^F]FDG-PET readouts into the core bvAD research criteria but only added them as supportive features. Fourth, evidence of amyloid-β pathology provided by PET, cerebrospinal fluid, or plasma biomarkers can upgrade the diagnosis from clinical bvAD to possible bvAD. Positive amyloid-β biomarkers substantially increase the likelihood that AD is the primary causative mechanism, but given their limited specificity, the possibility of amyloid-β as comorbid pathology cannot be ruled out, especially in older individuals and those who carry *APOE*ε4.^38,101^ The addition of biomarker evidence for tau pathology further increases the certainty for a bvAD diagnosis (ie, probable bvAD). Here, we make the distinction between biofluid and neuroimaging markers of tau pathology. For cerebrospinal fluid and plasma biomarkers of tau pathology, the differential diagnostic value for distinguishing AD from bvFTD is less well established, and as with amyloid-β markers, they become abnormal relatively early in the disease course, which lowers their specificity.^102,103^ Hence, a full AD-like fluid biomarker profile with abnormalities in both amyloid-β and phosphorylated tau supports a level II diagnosis of possible bvAD. Instead, the currently most widely used tau PET ligands (ie, [^18^F]flortaucipir, [^18^F]MK6240, and [^18^F]RO948) have consistently shown to bind selectively and with high affinity to the tau aggregates formed in AD (ie, combinations of 3R/4R tau in paired helical filaments), while neocortical tau PET uptake in sporadic bvFTD is negligible, resulting in excellent discriminative accuracy between AD and bvFTD.^104,105^ Furthermore, since tau PET uptake in the neocortex almost exclusively occurs in individuals positive for amyloid-β^104,106^ we consider a level I diagnosis (clinical bvAD) plus tau PET–positive results in an AD-like pattern^107^ supportive of a level III diagnosis of probable bvAD. Given the rapid developments in the blood-based biomarker field, the current distinction between neuroimaging and biofluid markers should be reevaluated in the future. Fifth, although the question of whether bvAD and dysexecutive AD exist on a single continuum or represent distinct clinical entities is yet unresolved, we deliberately developed criteria specific to bvAD. This was motivated by our previous study showing that only approximately 25% of bvAD cases additionally met dysexecutive AD criteria^16^; hence, bvAD occurs in isolation in most cases, as well as a recent article^12^ proposing specific dysexecutive AD criteria that explicitly exclude behavioral features. Therefore, while dysexecutive AD is considered if dysexecutive functioning and positive AD biomarkers are present in the absence of behavioral deficits, a diagnosis of bvAD is established when early behavioral alterations are observed in conjunction with either memory or executive functioning deficits and positive AD biomarkers (eFigure 8 in the Supplement).

### Limitations

There are several limitations. First, bvAD is a rare AD phenotype that, for the most part, has been described in single case studies and case series. The bvAD literature therefore consists of relatively few cohort studies generally characterized by modest sample sizes, which resulted in reduced statistical power to detect differences between bvAD vs bvFTD and tAD. This was further complicated by substantial heterogeneity in patient samples and outcome measures and subsequent substantial risk of bias across studies. Second, the variability across neuroimaging studies did not allow a meta-analytical approach; hence, we interpreted this literature using a systematic review. Third, in the behavioral, cognitive, and neuropathological meta-analyses, we combined comparable yet distinct study outcome measures, such as different neuropsychological tests for memory and executive functions, questionnaires for neuropsychiatric/behavioral features, or staining methods and selection of brain regions for histopathological assessment of amyloid-β and tau. Fourth, we did not account for possible copathologies (eg, Lewy bodies) that may contribute to the clinical phenotype. Fifth, the classification of possible bvAD and probable bvAD may be influenced by inherent differences in diagnostic accuracy of various amyloid and tau PET tracers, as well as assays for cerebrospinal fluid and plasma analysis, and centers likely vary in the reliability of their biomarker result interpretation. Sixth, there were only limited data on behavioral presentations of AD in diverse populations.

### Future Directions

Akin to the development of diagnostic criteria for posterior cortical atrophy, we consider the currently proposed research criteria as a stepping stone toward internationally established consensus criteria for bvAD. For posterior cortical atrophy, research criteria were first proposed by 2 research groups and were subsequently applied by other groups to establish a posterior cortical atrophy diagnosis for several years,^108,109^ followed by widely supported formal diagnostic criteria based on consensus by an international working group.^10^ Similarly, our bvAD criteria should improve the consistency and reliability of future research and possibly aid in the clinical assessment of bvAD, which in turn would enhance the diagnostic accuracy of future bvAD criteria to be established by a working group of worldwide experts. There are several promising novel biomarkers and behavioral features that could be included in future bvAD criteria, such as more objective measurements of behavior, such as social cognition in conjunction with biometric information (eg, eye tracking, face reading, galvanic skin response)^110^ or blood-based biomarkers of AD pathology (eg, phosphorylated tau, amyloid-β) and neurodegeneration (eg, neurofilament light chain).^111^ Furthermore, the diagnostic utility of potential bvAD-specific features (eg, relatively preserved disease insight, presence of hallucinations, and delusions) or measures of disease severity (eg, the frontotemporal lobar degeneration-modified Clinical Dementia Rating scale^112^) should be further investigated.

## Conclusions

Although the existence of bvAD is acknowledged in the most recent diagnostic and research criteria for AD dementia,^8,9^ there currently does not exist a set of criteria that provide specific recommendations for the diagnosis of bvAD. Our systematic review and meta-analyses of the current bvAD literature indicate that bvAD is clinically most similar to bvFTD, while it shares most pathophysiological features with tAD. Based on these insights, we provide the first research criteria for bvAD aimed at improving the consistency and reliability of future research and potentially facilitating clinical assessment of bvAD.

## Notes

Supplement. **eTable 1.** Full database queries **eTable 2.** Selection of frontal regions in autopsy studies **eTable 3. **Risk of bias assessment **eTable 4. **Characteristics of included studies **eTable 5. **Percentage of bvFTD features and NPI items in bvAD, bvFTD and tAD **eTable 6. **Functional connectivity and white matter hyperintensities in bvAD **eFigure 1.** Funnel plots for behavioral/neuropsychiatric data in meta-analysis **eFigure 2. **Funnel plots for cognitive data in meta-analysis **eFigure 3. **Funnel plots for neuropathological data in meta-analysis **eFigure 4. **Risk of bias assessment summary **eFigure 5. **Flow chart of study inclusion **eFigure 6. **Results of meta-analysis for behavioral and neuropsychiatric separately **eFigure 7. **Meta-analyses for neuropathological data in bvAD vs typical AD **eFigure 8. **Differences and overlap between bvAD and dysexecutive AD **eReferences. **Reference list Supplement
