# Supplementary material for: Research Criteria for the Behavioral Variant of Alzheimer Disease: A Systematic Review and Meta-analysis
Source: JAMA Neurol. 2021 Dec 6;79(1):48–60. doi: 10.1001/jamaneurol.2021.4417 (PMC8649917; doi:10.1001/jamaneurol.2021.4417)
Supplement: Supplement. — eTable 1. Full database queries eTable 2. Selection of frontal regions in autopsy studies eTable 3. Risk of bias assessment eTable 4. Characteristics of included studies eTable 5. Percentage of bvFTD features and NPI items in bvAD, bvFTD and tAD eTable 6. Functional connectivity and white matter hyperintensities in bvAD eFigure 1. Funnel plots for behavioral/neuropsychiatric data in meta-analysis eFigure 2. Funnel plots for cognitive data in meta-analysis eFigure 3. Funnel plots for neuropathological data in meta-analysis eFigure 4. Risk of bias assessment summary eFigure 5. Flow chart of study inclusion eFigure 6. Results of meta-analysis for behavioral and neuropsychiatric separately eFigure 7. Meta-analyses for neuropathological data in bvAD vs typical AD eFigure 8. Differences and overlap between bvAD and dysexecutive AD eReferences. Reference list Supplement [file jamaneurol-e214417-s001.pdf]

Supplemental Online Content

Ossenkoppele R, Singleton EH, Groot C, et al. Research criteria for the behavioral variant of alzheimer disease: a systematic review and meta-analysis. *JAMA Neurol*. Published online December 6, 2021. doi:10.1001/jamaneurol.2021.4417

eTable 1. Full database queries

eTable 2. Selection of frontal regions in autopsy studies

eTable 3. Risk of bias assessment

eTable 4. Characteristics of included studies

eTable 5. Percentage of bvFTD features and NPI items in bvAD, bvFTD and tAD

eTable 6. Functional connectivity and white matter hyperintensities in bvAD

eFigure 1. Funnel plots for behavioral/neuropsychiatric data in meta-analysis

eFigure 2. Funnel plots for cognitive data in meta-analysis

eFigure 3. Funnel plots for neuropathological data in meta-analysis

eFigure 4. Risk of bias assessment summary

eFigure 5. Flow chart of study inclusion

eFigure 6. Results of meta-analysis for behavioral and neuropsychiatric separately

eFigure 7. Meta-analyses for neuropathological data in bvAD vs typical AD

© 2021 Ossenkoppele R et al. *JAMA Neurology*

eFigure 8. Differences and overlap between bvAD and dysexecutive AD

eReferences. Reference list Supplement

This supplemental material has been provided by the authors to give readers additional information about their work.

© 2021 Ossenkoppele R et al. *JAMA Neurology*

eTable 1. Full database queries used in the present study

**Database**	**Search no.**	**Search terms**	**No. of studies**
PubMed/medline	1	(Alzheimer*[Title]) AND (behavio* variant[Title] OR executive variant[Title] OR dysexecutive variant[Title] OR behavio*/dysexecutive AD[Title] OR frontal variant[Title] OR frontal presentation[Title] OR nonamnestic[Title] OR non-amnestic[Title] OR heterogene*[Title] OR atypical[Title])	492
	2	(frontotemporal dementia[Title]) AND (pathology[Title] OR clinicopathologic* [Title])	73
Web of Science	1	TITLE: (Alzheimer*) AND TITLE: (behavio* variant OR executive variant OR dysexecutive variant OR behavio*/dysexecutive AD OR frontal variant OR frontal presentation OR nonamnestic OR non-amnestic OR heterogene* OR atypical)	581
	2	TITLE: (frontotemporal dementia) AND TITLE: (pathology OR clinicopathologic*)	111

© 2021 Ossenkoppele R et al. *JAMA Neurology*

eTable 2. Selection of frontal regions in autopsy studies

**Autopsy study**	**Frontal Subregion**
Balasa et al. 2011[1]	Not specified
Blennerhassett et al. 2014[2]	Randomly selected strips, perpendicular to the pial surface and spanning the cortical ribbon to the grey-white junction
Phillips et al. 2018[3]	Middle frontal gyrus
Singleton et al. 2021[4]	Frontal pole

© 2021 Ossenkoppele R et al. *JAMA Neurology*

eTable 3. Risk of bias assessment per domain according to the ROBINS-I tool per study included in the meta-analyses.

Study	D1 Bias due to confounding	D2 Bias in selection of participants into the study	D3 Bias in classification of interventions	D4 Bias due to deviations from intended interventions	D5 Bias due to missing data	D6 Bias in measurement of outcomes	D7 Bias in selection of the reported result	Overal bias
Woodward et al. 2010[5]	Y	Y	NA	NA	PY	N	N	Serious risk of bias
Balasa et al. 2011[1]	PY	PY	NA	NA	N	N	N	Moderate risk of bias
de Souza et al. 2013[6]	Y	PN	NA	NA	N	N	N	Moderate risk of bias
Mendez et al. 2013[7]	Y	PN	NA	NA	N	N	N	Moderate risk of bias
Fernández-Calvo et al. 2013[8]	PY	PY	NA	NA	PY	N	N	Moderate risk of bias
Blennerhassett et al. 2014[2]	PY	PN	NA	NA	PN	N	N	Moderate risk of bias
Ossenkoppele et al. 2015[9]	PN	PN	NA	NA	PN	N	N	Moderate risk of bias
Phillips et al. 2018[3]	PN	PY	NA	NA	PN	PY	N	Moderate risk of bias
Sala et al. 2020[10]	Y	PN	NA	NA	PY	N	PY	Moderate risk of bias
Therriault et al. 2020[11]	PN	PY	NA	NA	PY	N	N	Moderate risk of bias
Bergeron et al. 2020[12]	PY	Y	NA	NA	PY	PN	PY	Moderate risk of bias
Singleton et al. 2021[4]	PN	PN	NA	NA	PN	PY	N	Moderate risk of bias
Lehingue et al. 2021[13]	PY	Y	NA	NA	PY	PN	N	Serious risk of bias

Y=yes, *N*=no, *PY*=possible yes, *PN*=possible no.

© 2021 Ossenkoppele R et al. *JAMA Neurology*

eTable 4. Characteristics of included studies in chronological order

**Study**	**Design**	**Country**	**N**	**Participants**	**Controls**	**Age**	**Sex**	**MMSE**	**Confirmation of AD**	**Main topic of group study**	**Type of data in case studies**					
											**CL**	**CO**	**SO**	**NI**	**PA**	**GE**
Brun et al. 1976[14]	Case study	Sweden	5	bvAD	-	56 (5.69)	60	n/a	Autopsy		X				X	
Shibayama et al. 1978[15]	Case study	Japan	1	bvAD	-	70	0	n/a	Autopsy		X				X	
Shuttleworth 1984[16]	Case study	US	2	bvAD	-	49 (3)	0	n/a	No		X	X		X		
Brun 1987[17]	Case study	Sweden	2	bvAD	-	75 (6)	100	n/a	Autopsy		X				X	
Perani et al. 1988[18]	Case study (within a cross-sectional observational study)	Italy	1	bvAD	-	56	100	n/a	No		X	X		X		
Bird et al. 1989[19]	Cross-sectional observational study	US	2	bvAD	-	66 (1)	0	n/a	Autopsy		X				X	X
Grady et al. 1990[20]	Cross-sectional observational study	US	5	bvAD	Subgroups of AD	71.5	20	8 (7)	No	Neuroimaging						
Molchan et al. 1990[21]	Case study	US	2	bvAD	-	58 (3)	50	12.5 (0.5)	No		X	X				

© 2021 Ossenkoppele R et al. *JAMA Neurology*

Raux et al. 2000[22]	Case series	France	3	bvAD	-	49.3 (10.4)	100	n/a	Genetic							
Rippon et al. 2003[23]	Case study	US	2	bvAD	-	43.5 (1.5)	0	18.5 (5.5)	Genetic		X	X		X	X	X
Yokota et al. 2003[24]	Case study	Japan	3	bvAD	-	33.7 (4.5)	66.7	n/a	Autopsy		X				X	X
Doran & Larner 2004[25]	Case study		2	bvAD	-	49 (0)	50	n/a	Genetic		X				X	X
Kertesz et al. 2005[26]	Cross-sectional observational cohort study	Canada	1	bvAD	-	n/a	n/a	n/a	Autopsy	Clinicopatholo gical						
Shi et al. 2005[27]	Cross-sectional cohort study	China	1	bvAD	-	59	0	n/a	Autopsy	Clinicopatholo gical						
Forman et al. 2006[28]	Cohorts study	US	19	bvAD	FTLD	60.3	47	20.1 (2-29)	Autopsy	Clinicopatholo gical						
Larner 2006[29]	Case study	UK	2	bvAD	-	54 (2)	0	16.5 (0.5)	No		X	X		X		X
Alladi et al. 2007[30]	Corss-sectional observational cohort study	UK	2	bvAD	atypical AD	n/a	n/a	n/a	Autopsy	Clinicopatholo gical						
Rabinovi ci et al. 2007[31]	Cross-sectional observational study	US	2	bvAD	-	54 (1)	50	22.5 2.5	Amyloid PET	Neuroimaging						
Snowden et al. 2007[32]	Cross-sectional observational cohort study	UK	12	bvAD	AD & atypical AD	49 (8)	25	n/a	No	Cognitive & Genetic						

© 2021 Ossenkoppele R et al. *JAMA Neurology*

Taylor et al. 2008[33]	Case study	UK	1	bvAD	-	66	0	28	Autopsy		X	X		X	X	
Kile et al. 2009[34]	Case study	US	1	bvAD	-	n/a	0	30	Autopsy							
Bigio et al. 2010[35]	Cross-sectional observational study	US	10	bvAD	AD & FTD	58 (6.5)	30	n/a	Autopsy		X				X	
Habek et al. 2010[36]	Case study	Croatia	1	bvAD	-	56	0	n/a	Biopsy		X			X		
Lehman et al. 2010[37]	Cross-sectional observational study	UK	2	bvAD	AD & FTD	59 (1.4)	50	9.5 (0.7)	Autopsy	Clinicopathological & Neuroimaging						
Piscopo et al. 2010[38]	Case study	Italy	1	bvAD	-	63	n/a	11	Genetic		X	X				X
Woodward et al. 2010[5]	Cross-sectional observational cohort study	Canada	18	bvAD	AD & FTD	74.7 (7)	44.4	18.6 5.9	No	Clinical & Genetic						
Balasa et al. 2011[1]	Cross-sectional observational cohort study	Spain	7	bvAD	AD & atypical AD	55.6 (3.7)	28.6	n/a	Autopsy	Clinicopathological & Genetic						
Rabinovici et al. 2011[39]		US	3	bvAD	AD, FTD & CN	n/a	n/a	n/a	PET	Neuroimaging						
Snowden et al. 2011[40]	Cross-sectional observational study	UK	2	bvAD	-	60.5 (6.5)	50	n/a	Autopsy	Clinicopathological						

© 2021 Ossenkoppele R et al. *JAMA Neurology*

Whitwell et al. 2011[41]	Cross-sectional observational study	US	3	bvAD	AD & CN	58.33 (3.3)	33.3	n/a	Autopsy	Neuroimaging						
Borroni et al. 2012[42]	Case study	Italy	1	bvAD	-	68	0	21	Genetic & CSF							
Duker et al. 2012[43]	Case study	US	1	bvAD	-	58	0	n/a	No		X	X		X		
Wallon et al. 2012[44]	Case series	France	8	bvAD	-	n/a	n/a	n/a	Genetic	Genetic						
De Souza et al. 2013[6]	Case series	France	8	bvAD	AD, bvFTD & CN	63.5 (8.9)	12.5	17.6 5.6	CSF	Cognitive & Neuroimaging						
Fernandez-Calvo et al. 2013[8]	Cross-sectional observational study	Spain	13	bvAD	AD & CN	72.8 (7.6)	31	22.5 2.1	No	Cognitive & Neuropsychiatric						
Herrero-San Martin et al. 2013[45]	Case study	Spain	2	bvAD	-	56 (4)	50	n/a	Autopsy		X				X	
Marini et al. 2013[46]	Case study	Italy	1	bvAD	-	59	100	n/a	Genetic							
Mendez et al. 2013[46]	Cross-sectional observational cohort study	US	21	bvAD	FTLD	69.3 (8.3)	14.3	13.3 9.4	Autopsy	Clinicopathological						

© 2021 Ossenkoppele R et al. *JAMA Neurology*

Blennerhassett et al. 2014[2]	Cross-sectional observational study	Australia	6	bvAD	AD & bvFTD	68 (14)	33.4	n/a	Autopsy	Pathological						
Leger et al. 2014[47]	Cross-sectional observational study	US	31	bvAD	FTLD	n/a	n/a	n/a	Autopsy	Clinicopathological						
Nijgaard et al. 2014[48]	Case study	US	1	bvAD	-	52	0	30	Genetic		X	X		X		X
Balasa et al. 2015[49]	Cross-sectional observational study	Spain	13	bvAD	FTLD	n/a	n/a	n/a	Autopsy	Clinicopathological						
Ossenkoppele et al. 2015[9]	Cross-sectional observational study	Netherlands & US	55	bvAD	AD, bvFTD & CN	64.7 (8.8)	27.3	22.5 5.4	CSF/PET/ Autopsy	Clinical, Cognitive & Neuroimaging						
Paterson et al. 2015[50]	Cross-sectional observational study	UK	8	bvAD	AD, atypical AD & CN	61.5 (6.4)	62.5	17.4 6.1	CSF/PET/ autopsy	Cerebrospinal fluid						
Woodward et al. 2015[51]	Cross-sectional observational study	NA	13	bvAD	AD	81.6 (4.1)	38.5	23.9	No	Neuroimaging						
Li et al. 2016[52]	Case study	China	1	bvAD	bvFTD	71	100	15	PET		X	X		X		X
Ossenkoppele et al. 2016[53]	Cross-sectional observational study	US	1	bvAD	AD, atypical AD & CN	59	0	21	PET							
Scialo et al. 2016[54]	Case study	Italy	1	bvAD	-	68	100	27	PET		X	X		X		X

© 2021 Ossenkoppele R et al. *JAMA Neurology*

Dickerson et al. 2017[55]	Case series	US	1	bvAD	-	62	100	n/a	CSF		X	X		X		
Duclos et al. 2017[56]	Case study	France	1	bvAD	CN	60	100	n/a	CSF		X	X	X	X		
Kawakatsu et al. 2017[57]	Case series	Japan	3	bvAD	-	57.7 (1.3)	33.3	n/a	Autopsy		X			X	X	
Oboudiyat et al. 2017[58]	Cross-sectional observational study	US	2	bvAD	-	n/a	n/a	n/a	CSF & autopsy	Cerebrospinal fluid						
Perry et al. 2017[59]	Cross-sectional observational study	US	15	bvAD	FTLD	62.8 (43-83)	33.3	19.8 6.9	Autopsy	Clinicopathological & Neuroimaging						
Rawtaer et al. 2017[60]	Case study	Canada	1	bvAD	-	68	0	11	No		X	X		X		X
Sawyer et al. 2017[61]	Case series	US	3	bvAD	-	76.3 (3.1)	33.3	n/a	Autopsy		X	X		X	X	
Bagyinskzy et al. 2018[62]	Case series	Korea	1	bvAD	-	41	100	24	Genetic		X	X		X		X
Boon et al. 2018[63]	Cross-sectional observational study	Netherlands	3	bvAD	AD	60.7 (1.3)	0	n/a	Autopsy	Pathological						
Phillips et al. 2018[3]	Observational cross-sectional study	US	22	b/dAD	AD, atypical AD & CN	64.3 (8.2)	50	19.6 8.4	CSF/autopsy	Neuroimaging & Pathological						

© 2021 Ossenkoppele R et al. *JAMA Neurology*

Seo et al. 2018[64]	Retrospective observational study	US	23	bvAD	-	n/a	n/a	n/a	Autopsy	Clinicopathological						
Whitwell et al. 2018[65]	Cross-sectional observational study	US	6	b/dAD	AD & atypical AD	n/a	n/a	n/a	PET	Neuroimaging						
De Souza et al. 2019[66]	Case study	Brazil	1	bvAD	-	68	100	29	CSF		X	X	X	X		
Foiani et al. 2019[67]	Cross-sectional observational study	UK	2	bvAD	FTLD	n/a	n/a	n/a	CSF	Cerebrospinal fluid						
Monacelli et al. 2019[68]	Case study	Italy	1	bvAD	-	60	100	25	Genetic		X	X		X		X
Nolan et al. 2019[69]	Cross-sectional observational study	US	5	bvAD	AD, atypical AD & CN	66.2 (4.8)	20	n/a	Autopsy	Pathological						
Pawlowski et al. 2019[70]	Cross-sectional observational study	Germany	8	bvAD	AD & FTD	n/a	n/a	n/a	CSF	Clinical & Cerebrospinal fluid						
Phillips et al. 2019[71]	Cross-sectional & longitudinal observational study	US	12	bvAD	AD, atypical AD & CN	63.9 (59.7-69.5)	41.7	23 (17-26)	CSF/autopsy	Neuroimaging						
Pillai et al. 2019[72]	Cross-sectional observational study	US	4	b/dAD	AD & atypical AD	n/a	n/a	n/a	CSF	Cerebrospinal fluid						

© 2021 Ossenkoppele R et al. *JAMA Neurology*

Tan et al. 2019[73]	Cross-sectional observational study	Australia	9	bvAD	AD	65 (10)	22	n/a	Autopsy	Pathological						
Wang et al. 2019[74]	Cross-sectional obersational study	China	13	b/dAD	AD, atypical AD & CN	68 (3.4)	69.2	17 (5.6)	PET	Neuroimaging						
Wong et al. 2019[75]	Case study	Australia	1	bvAD	AD, bvFTD & CN	57	0	n/a	PET		X	X	X	X		
Bergeron et al. 2020[12]	Cross-sectional observational study	Canada	8	b/dAD	Atypical AD & FTD	61.6	n/a	20.7	CSF/PET	Neuroimaging						
Cai et al. 2020[76]	Case study	China	1	bvAD	-	52	100	n/a	PET		X	X		X		X
Cousins et al. 2020[77]	Cross-sectional observational study	US	2	bvAD	AD, atypical AD, FTD & CN	n/a	n/a	n/a	Autopsy/ CSF	Clinicopathological & Cerebrospinal fluid						
Li et al. 2020[78]	Case study	Taiwan	1	bvAD	-	66	0	10	PET		X	X		X		X
Paquin et al. 2020[79]	Case study	Canada	1	bvAD	-	60	100	28	Tau and amyloid PET		X	X			X	
Sala et al. 2020[10]	Cross-sectional observational study	Italy	15	b/dAD	AD & atypical AD	62.47 (5.7)	33.3	16.5 (5.2)	CSF	Neuroimaging						
Scarioni et al. 2020[80]	Cross-sectional observational study	Netherlands	35	bvAD	FTLD	n/a	n/a	n/a	Autopsy	Clinicopathological						
Singleton et al. 2020[81]	Cross-sectional observational study	US	29	bvAD	AD, bvFTD & CN	64.4 (9.4)	41	22 (5.9)	CSF/PET/ autopsy	Neuroimaging						

© 2021 Ossenkoppele R et al. *JAMA Neurology*

Therriault et al. 2020[11]	Cross-sectional observational study	Canada	15	b/dAD	AD & CN	65.93 (8.8)	60	19.6 (5.3)	Tau & amyloid PET	Neuroimaging						
Bergeron et al. 2021[82]	Case series	Canada	8	bvAD	AD & bvFTD	59.5 (7.9)	25	22.3 (5.9)	CSF/PET	Cognivite & Neuropsychiatric & Neuroimaging						
Lehingue et al. 2021[13]	Cross-sectional prospective observational study	France	20	bvAD	AD & bvFTD	71.5 (66-76)	35	25 (21-26)	CSF	Cognitive & Neuropsychiatric & Neuroimaging						
Singleton et al. 2021[4]	Cross-sectional observational study	Netherlands, Sweden & US	7 & 8	bvAD	AD	69.1 (8.4) & 66.6 (6.0)	14.3 & 50.0	21.7 (2.8)	CSF/PET and autopsy	Neuroimaging & Pathological						
Zhu et al. 2021[83]	Case study	China	1	bvAD	-	63	0	3	CSF & PET		X	X		X		X

Numbers are depicted as mean (sd). *CL*=clinical, *COG*=cognition, *SOC*=social cognition, *NI*=neuroimaging, *PA*=pathological, *GEN*=genetic, *CSF*=cerebrospinal fluid, *PET*=positron emission tomography, AD=Alzheimer's disease, *bvAD*=behavioral variant of Alzheimer's disease, *bvFTD*=behavioral variant frontotemporal dementia.

© 2021 Ossenkoppele R et al. *JAMA Neurology*

eTable 5. Weighted mean percentage of patients with separate behavioral and neuropsychiatric symptoms in bvAD and bvFTD.

Diagnosis	bvAD	bvFTD	tAD	P-value of χ^2-test bvAD vs bvFTD	P-value of χ^2-test bvAD vs tAD
bvFTD criteria, n□	148†	313*			
Disinhibition	60.80	68.58		0.10	NA
Apathy	68.80	77.37		0.05	NA
Loss of empathy	54.64	53.64		0.83	NA
Compulsiveness	45.00	68.50		<0.00001*	NA
Hyperorality	35.89	64.11		<0.00001*	NA
NPI, n◊	52	156	1090▪		
Eating changes	41.33	44.64	31.4	0.57	0.12
Night-time behaviors	39.60	40.73	20.0	0.94	0.0003*
Irritability	50.81	42.15	42.9	0.33	0.32
Euphoria	16.62	27.09	6.0	0.16	0.005*
Anxiety	54.15	43.10	31.6	0.17	0.001*
Depression	34.19	35.10	32.1	0.93	0.78
Agitation	67.85	43.42	16.2	0.003*	<0.00001*
Hallucination	28.23	9.00	4.6	0.0003*	<0.00001*
Delusions	36.62	13.42	9.3	0.0003*	<0.00001*
Motor behavior	50.38	57.10	18.9	0.38	<0.00001*

bvAD=behavioral variant of Alzheimer's disease, *bvFTD*=behavioral variant frontotemporal dementia, *tAD*=typical Alzheimer's disease.

†Based on estimates from 7 group studies(de Souza et al. 2013[6], Mendez et al. 2013[7], Blennerhassett et al. 2014[2], Ossenkoppele et al. 2015[9], Perry et al. 2017[59], Leger et al. 2014[47], Phillips et al. 2019[71])
* Based on estimates from 4 group studies (Mendez et al. 2013[7], Ossenkoppele et al. 2015[9], Perry et al. 2017[59], Leger et al. 2014[47])
□ Percentages are based on percentage per symptoms assessed by NPI, clinical evaluation or chart reviews from studies specified above.
◊ Percentages are based on percentage per symptoms assessed by NPI from two studies (Mendez et al. 2013[7], Leger et al. 2014[47]).
▪ Based on a cohort of Aβ-positive AD dementia patients from the Amsterdam dementia cohort (Eikelboom et al.[84]).

© 2021 Ossenkoppele R et al. *JAMA Neurology*

eTable 6. Results of functional connectivity and white matter hyperintensities in bvAD

Study	Subjects	Age	Sex	MMSE	AD confirmation	Contrasts	Modality	Findings
**Functional connectivity**								
Wang et al. 2019[74]	13 b/dAD	68.0 (3.4)	30.77	17.0 (5.6)	PiB PET	38 typical AD, 20 CU	FDG-PET	The left executive control network showed the highest goodness-of-fit in both b/dvAD and tAD and no differences in PiB PET uptake in network templates was observed
Phillips et al. 2019[71]	12 bvAD	16.0 [13.5, 18.0]	58.3	23.0 [17.0, 26.0]	CSF/autopsy	17 typical AD	Diffusion MRI	Higher node degree predicted greater annualized grey matter volume loss in both bvAD and typical AD groups and bvAD showed a less negative slope of association between node degree and longitudinal atrophy than typical AD
Singleton et al. 2020[81]	29 bvAD	64.4 (9.4)	59.0	22.0 (5.9)	CSF/PET/autopsy	28 typical AD, 28 bvFTD, 34 CU	FDG-PET	The anterior default mode network showed highest goodness-of-fit in bvAD (tAD <bvAD=bvFTD), and significantly less metabolic connectivity of the posterior cingulate cortex to the (right) prefrontal cortex was observed in bvAD compared to tAD
**White matter hyperintensities**								
Singleton et al. 2020[81]	29 bvAD	64.4 (9.4)	59.0	22.0 (5.9)	CSF/PET/autopsy	28 typical AD, 28 bvFTD, 34 CU	FLAIR-MRI	In comparison to tAD, bvAD patients showed lower juxtacortical left temporal and subcortical WMHV and higher right temporal juxtacortical WMHV

*b/*dAD=behavioral/dysexecutive variant of Alzheimer's disease, *bvAD*=behavioral variant of Alzheimer's disease, *bvFTD*=behavioral variant frontotemporal dementia, *tAD*=typical Alzheimer's disease, *CU*=cognitively unimpaired individuals, *CSF*=cerebrospinal fluid, *PET*=positron emission tomography, *MRI*=magnetic resonance imaging.

© 2021 Ossenkoppele R et al. *JAMA Neurology*

eFigure 1. Funnel plots of meta-analyses of behavorial/neuropsychiatric data for behavioral variant AD versus typical AD and bvFTD.

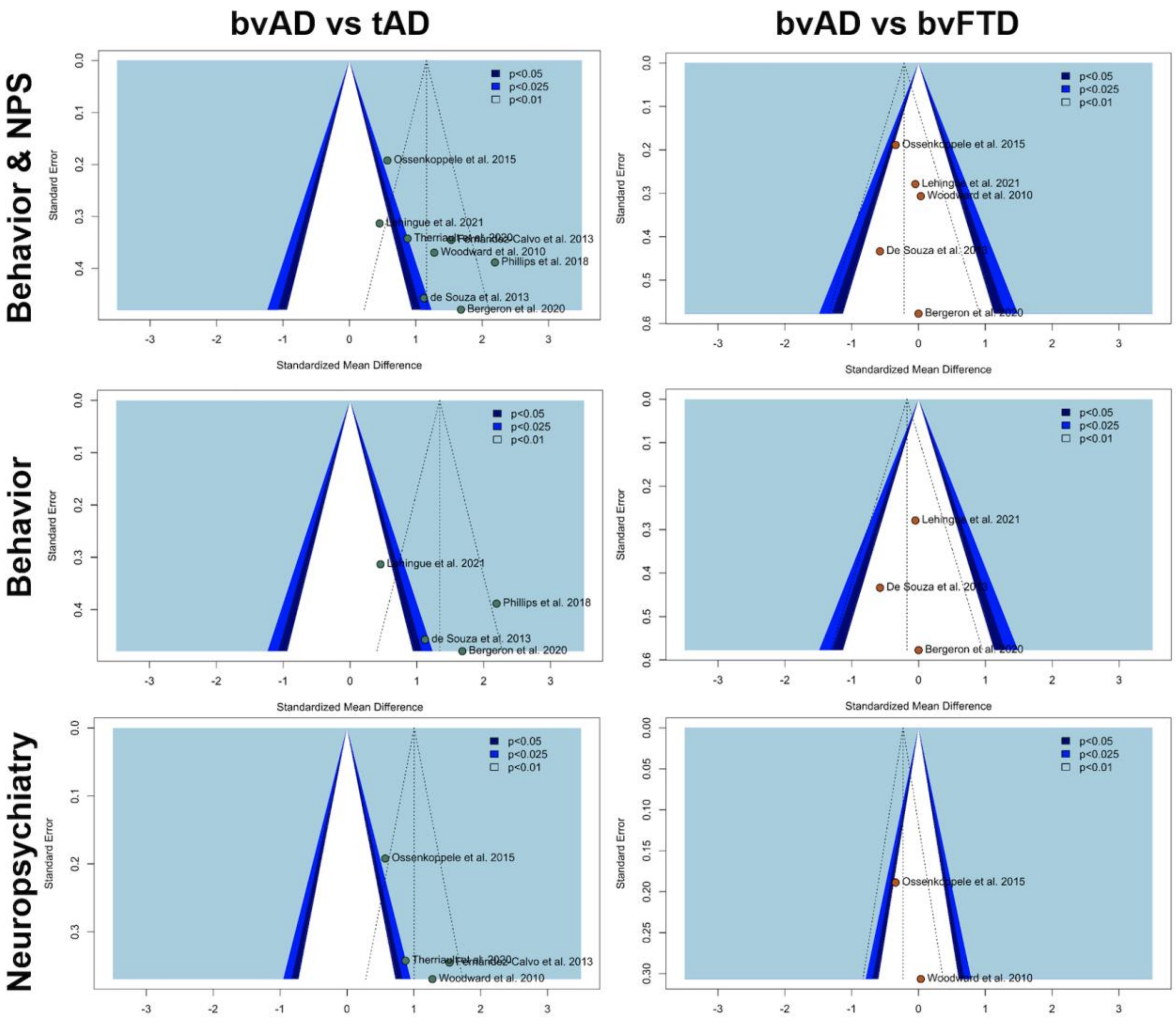

Funnel plots displaying the position of individual studies on their standardized mean difference (x-axis) relative to their standard error (y-axis). If no publication bias were present, studies would be aligned symmetrically within the dotted triangles, indicating symmetrical locations surrounding the mean effect size, with smaller studies at the lower ends of the plot and larger studies on the higher end of the plot.. The dark blue, medium dark blue and light blue parts represent the locations where the effect of the individual study is significant at $p<0.05$, $p<0.025$ and $p<0.01$ compared to the standardized mean difference at 0, whereas the dotted lines represent the mean effect size of the specific studies included. The current plots suggest a lower symmetrical tendency in bvAD vs tAD contrasts compared to bvAD vs bvFTD contrasts, indicating higher publication bias in the bvAD vs tAD contrasts, although the number of studies and sample sizes were small.

© 2021 Ossenkoppele R et al. *JAMA Neurology*

eFigure 2. Funnel plots of meta-analyses for behavioral and neuropsychiatric symptom data separately for bvAD vs typical AD and bvFTD.

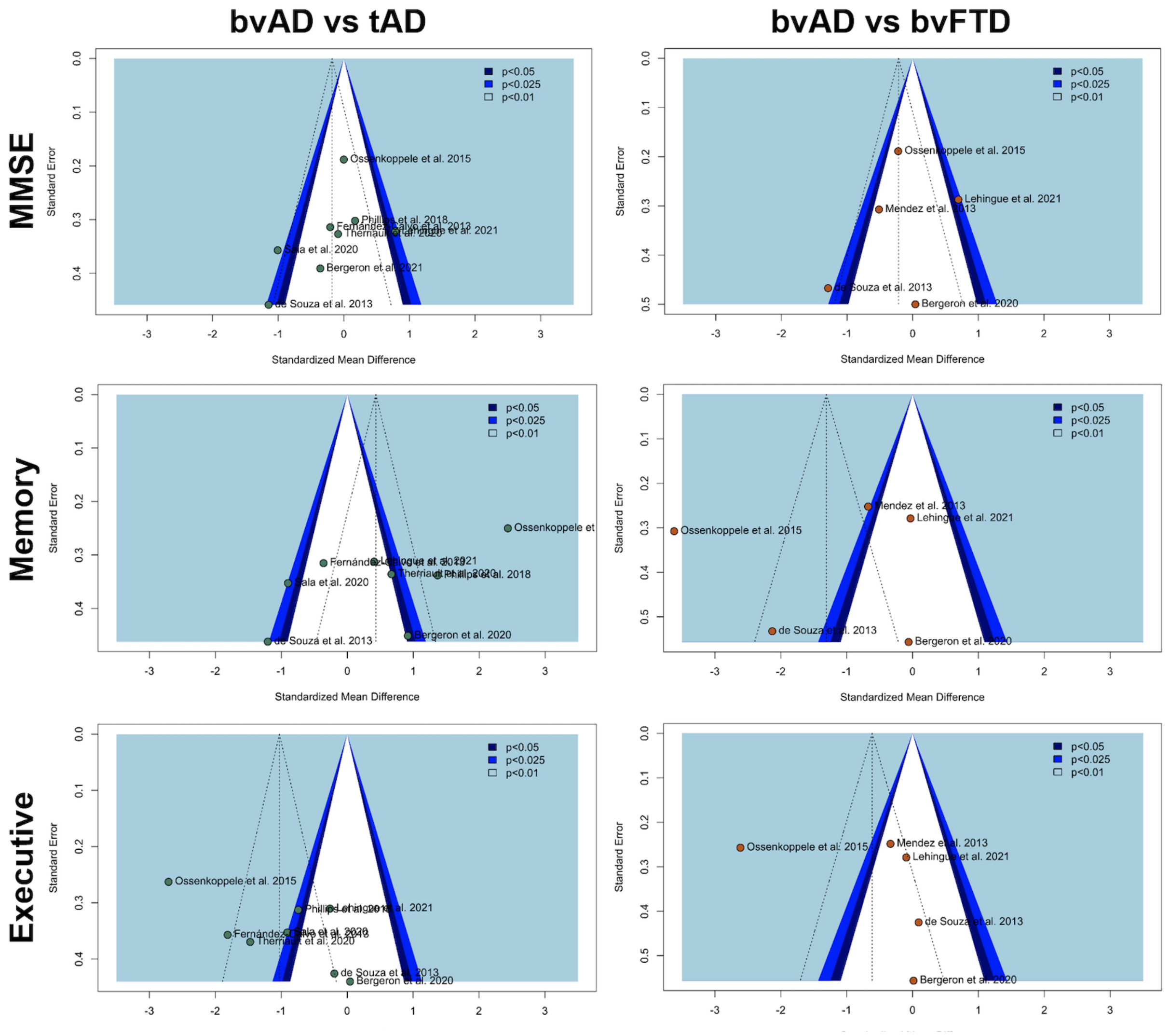

Funnel plots displaying the position of individual studies on their standardized mean difference (x-axis) relative to their standard error (y-axis). If no publication bias were present, studies would be aligned symmetrically within the dotted triangles, indicating symmetrical locations surrounding the mean effect size, with smaller studies at the lower ends of the plot and larger studies on the higher end of the plot. The dark blue, medium dark blue and light blue parts represent the locations where the effect of the individual study is significant at $p<0.05$, $p<0.025$ and $p<0.01$ compared to the standardized mean difference at 0, whereas the dotted lines represent the mean effect size of the specific studies included. The current plots suggest a higher symmetrical tendency in the MMSE contrasts than in the memory and executive domains, indicating higher publication bias in the memory and executive functioning domains than in the MMSE, although the number of studies and sample sizes were small.

© 2021 Ossenkoppele R et al. *JAMA Neurology*

eFigure 3. Funnel plots of meta-analyses of neuropathological data in bvAD versus typical AD and bvFTD.

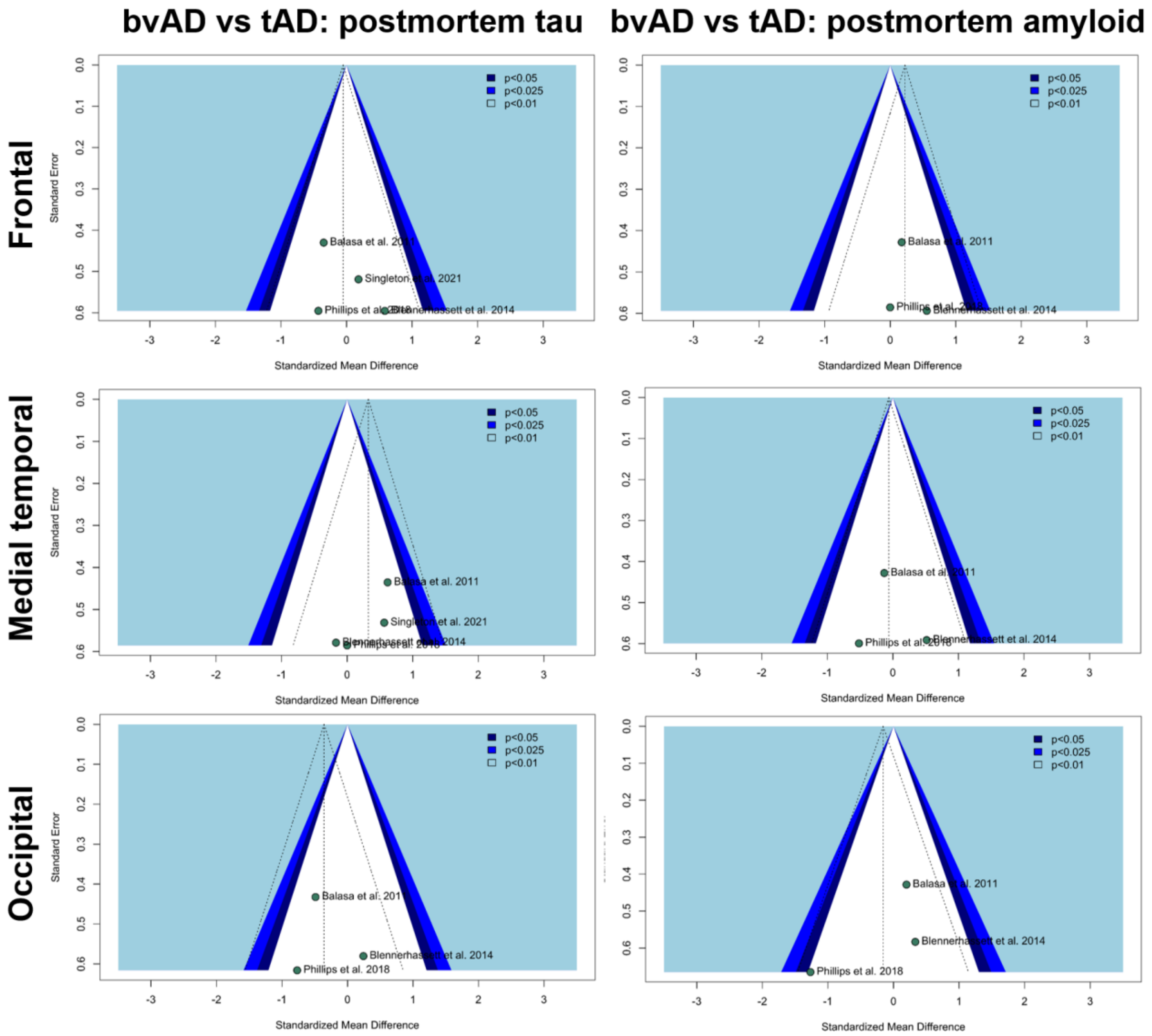

Funnel plots displaying the position of individual studies on their standardized mean difference (x-axis) relative to their standard error (y-axis). If no publication bias were present, studies would be aligned symmetrically within the dotted triangles, indicating symmetrical locations surrounding the mean effect size, with smaller studies at the lower ends of the plot and larger studies on the higher end of the plot. The dark blue, medium dark blue and light blue parts represent the locations where the effect of the individual study is significant at p<0.05, p<0.025 and p<0.01 compared to the standardized mean difference at 0, whereas the dotted lines represent the mean effect size of the specific studies included. Although few studies were included per plot, the current plots show an overall symmetrical tendency, marking marginal publication bias.

© 2021 Ossenkoppele R et al. *JAMA Neurology*

eFigure 4. Summary results of Risk of Bias assessment according to the ROBINS-I tool for studies included in the meta-analyses

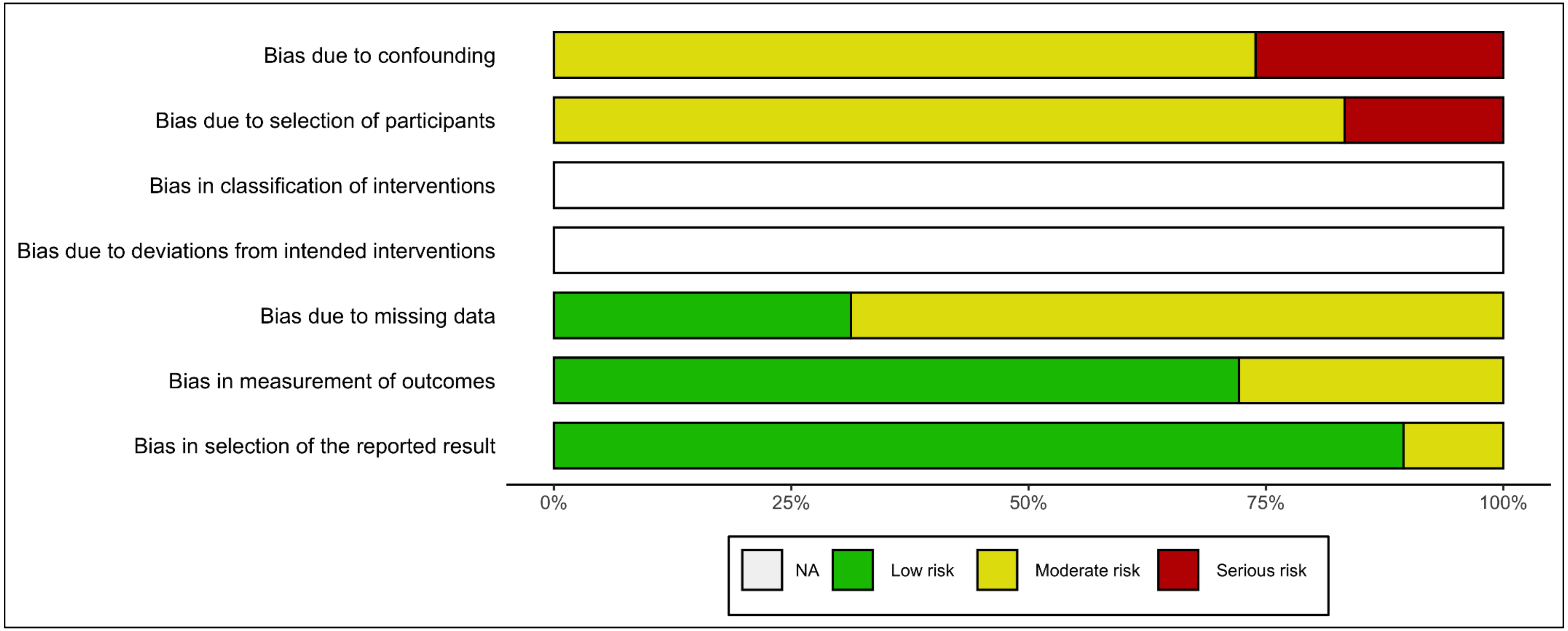

The ROBINS-I tool for non-randomized studies (https://www.riskofbias.info/) was applied to assess Risk of Bias across studies. Since the domains ‘Bias in classification of interventions’ and ‘Bias due to deviations from intended interventions’ were not applicable to the currently assessed studies, these were not filled out (*NA*=not available). See Table S3 for further details.

© 2021 Ossenkoppele R et al. *JAMA Neurology*

eFigure 5. Flow chart of study inclusion

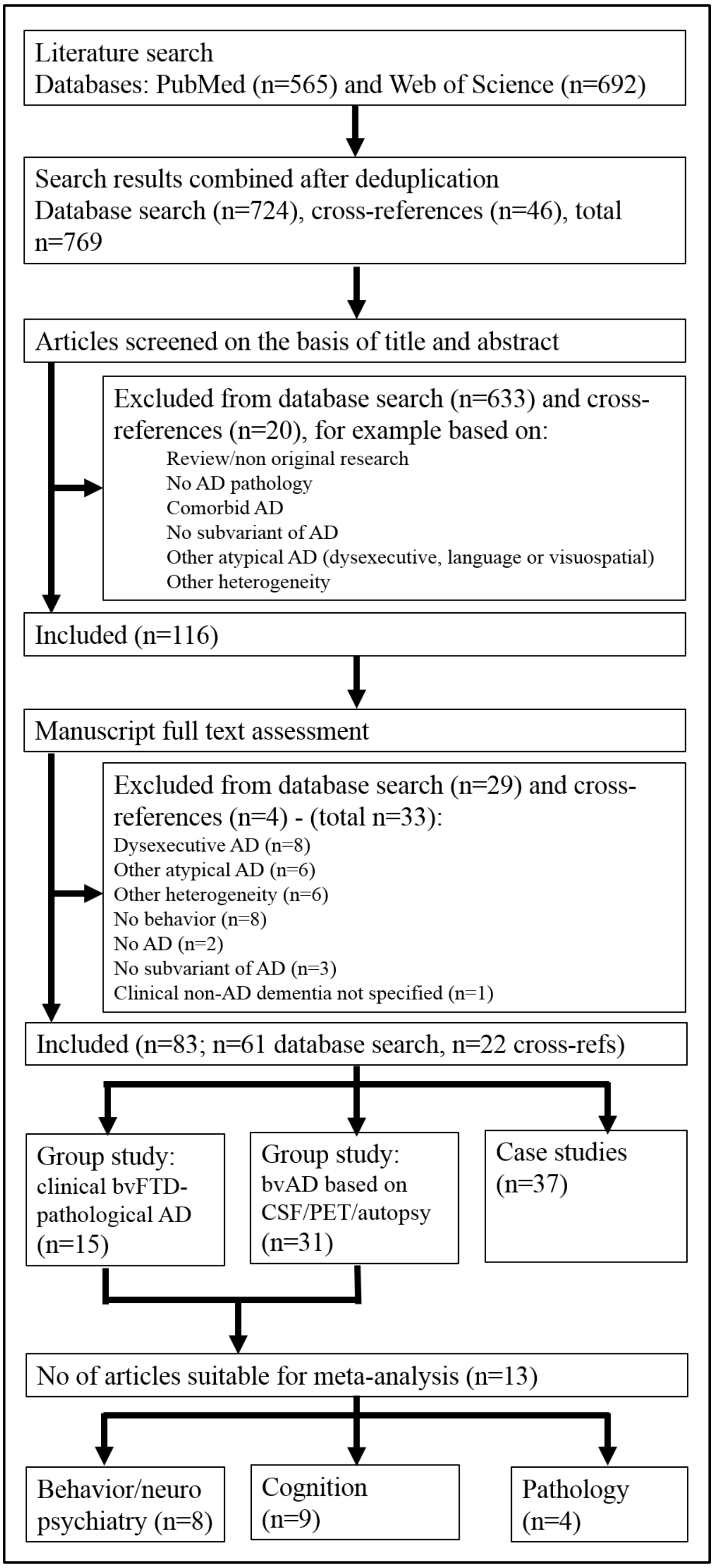

AD=Alzheimer's disease, *bvAD*=behavioral variant of AD, *bvFTD*=behavioral variant frontotemporal dementia, *CSF*=cerebrospinal fluid, *PET*=positron emission tomography.

© 2021 Ossenkoppele R et al. *JAMA Neurology*

eFigure 6. Meta-analyses for behavior and neuropsychiatric symptoms separately in bvAD vs typical AD and bvFTD

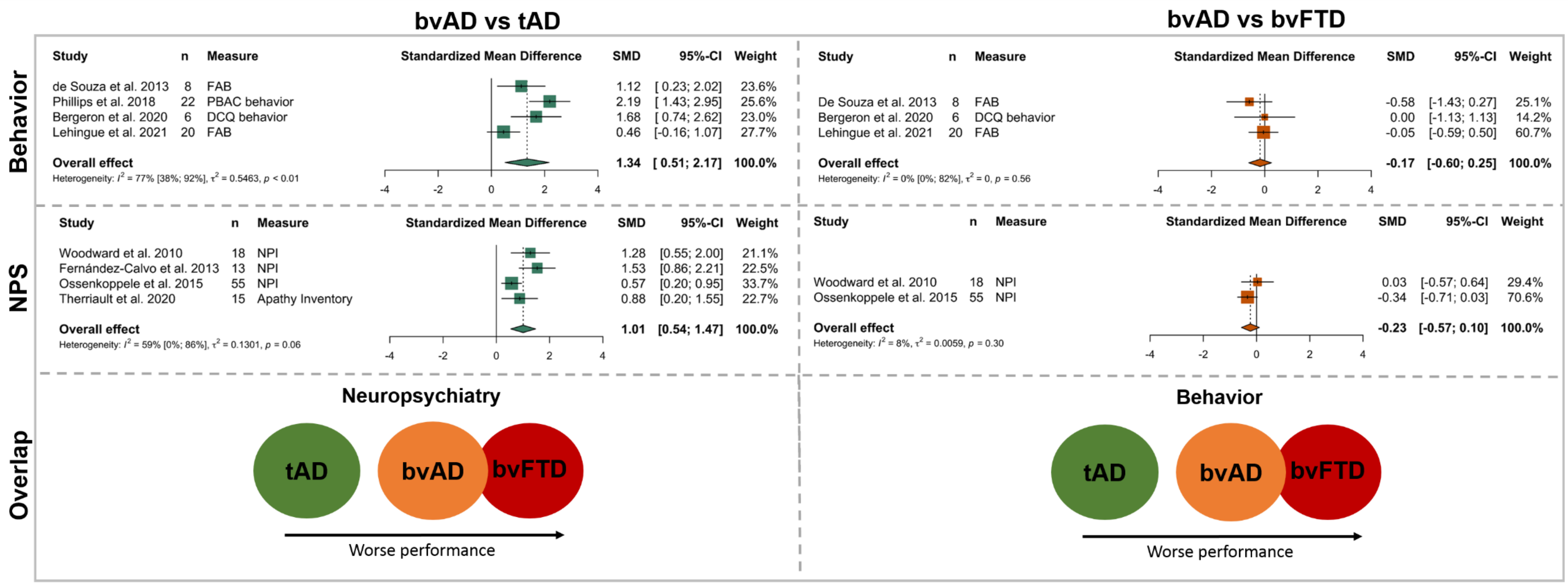

Plots showing meta-analysis results for behavior and neuropsychiatric symptoms separately between patient groups. These plots show similar scores in both behavioral as neuropsychiatric scales scores in bvAD versus bvFTD and a similar difference in behavioral and neuropsychiatric scale scores in bvAD versus typical AD. For all meta-analyses, positive standardized mean differences indicate a greater neuropathological burden in bvAD versus typical AD.

bvAD=behavioral variant of AD, *tAD*=typical AD, *bvFTD*=behavioral variant frontotemporal dementia, *FAB*=Frontal Assessment Battery, *PBAC*=Philadelphia Brief Assessment of Cognition, *DCQ*=Dépistage Cognitif de Québec, *SMD*=standardized mean difference.

© 2021 Ossenkoppele R et al. *JAMA Neurology*

eFigure 7. Meta-analyses for neuropathological data in bvAD vs typical AD

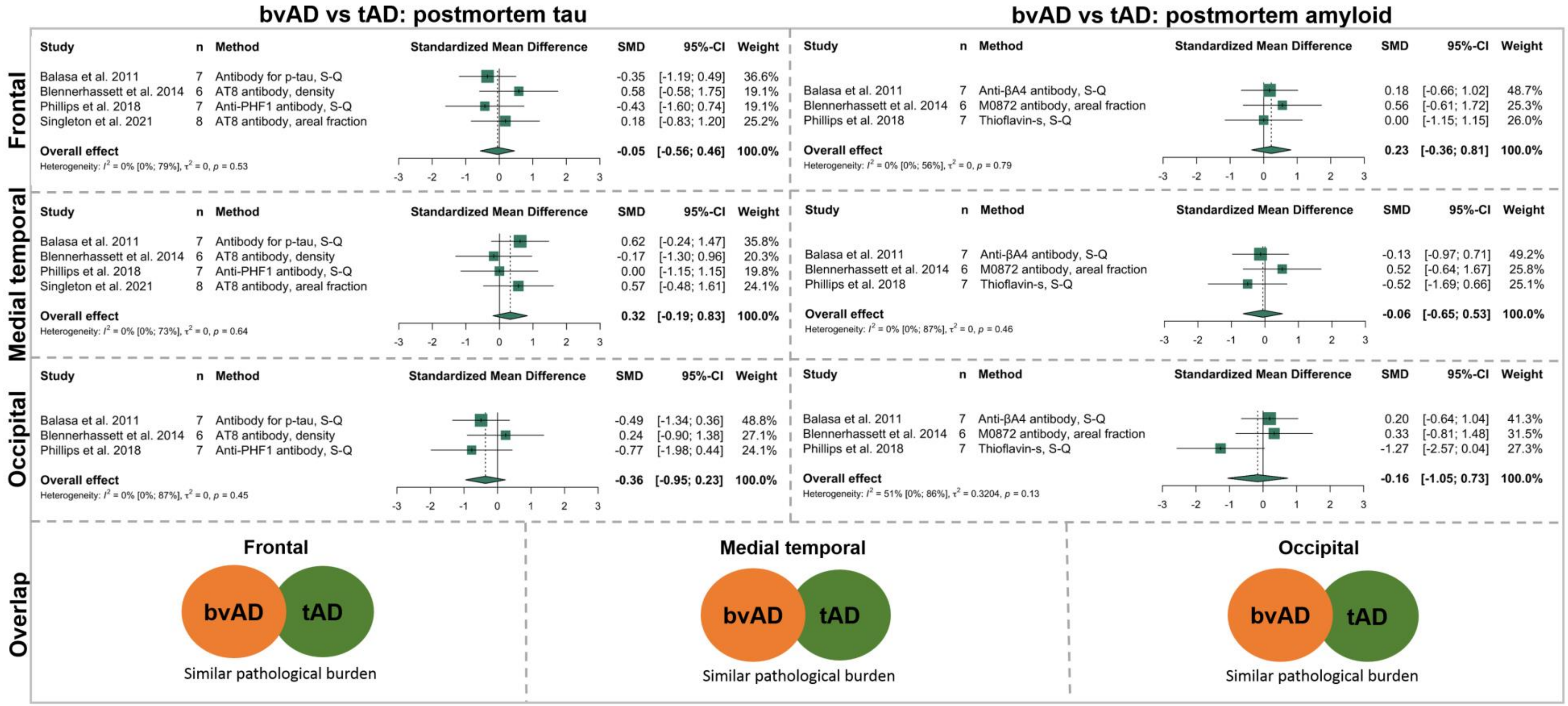

The figure shows results of meta-analyses across frontal (top row), medial temporal (middle row) and occipital (bottom row) regional quantification of postmortem tau (left column) and amyloid-β (right column) pathology in bvAD versus typical AD. Frontal regions included the frontal pole[4], middle frontal gyrus[3] and randomly selected frontal areas[2], and was not further specified in one study[1]. For all meta-analyses, positive standardized mean differences indicate a greater neuropathological burden in bvAD versus typical AD.

SMD=standardized mean difference, *S-Q*=semi-quantitative.

© 2021 Ossenkoppele R et al. *JAMA Neurology*

eFigure 8. Differences and overlap between bvAD and dysexecutive AD

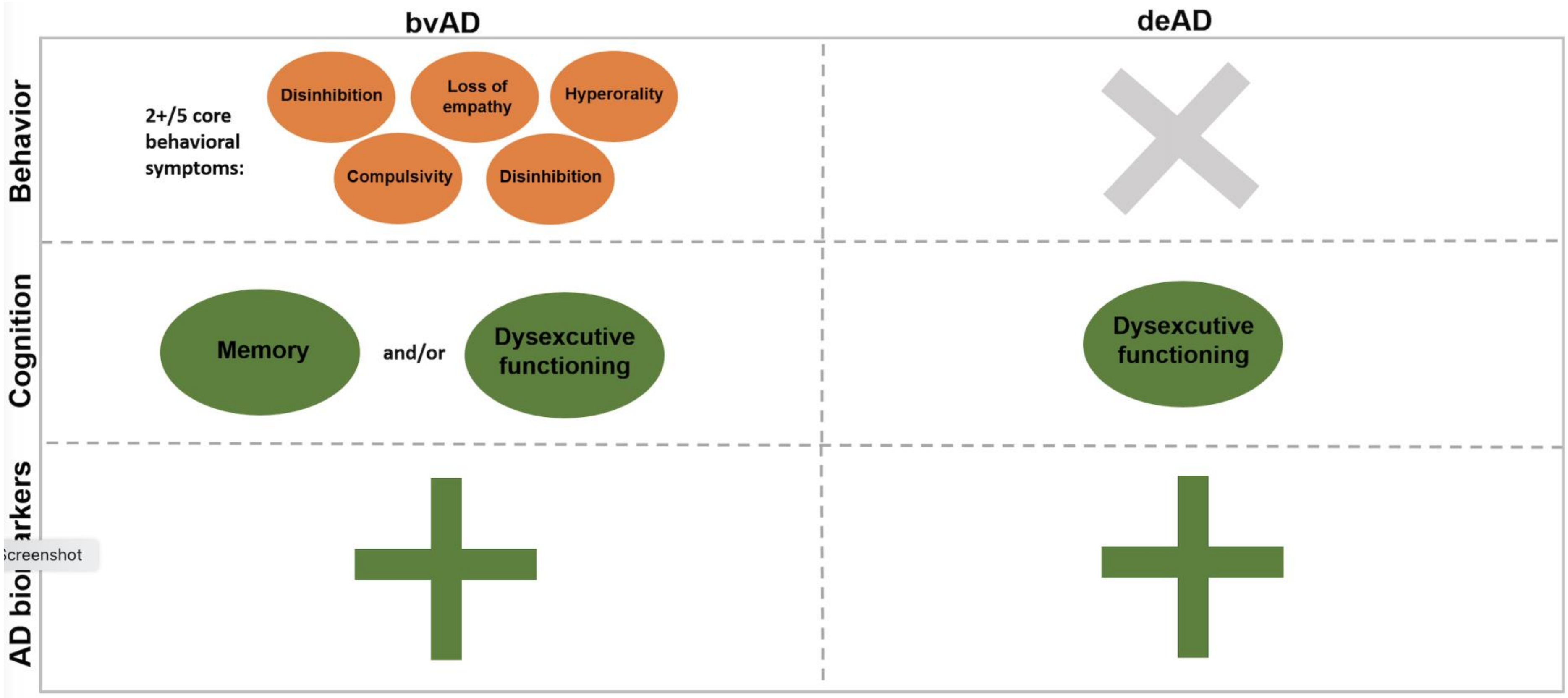

Differences and overlap between the behavioral variant of AD (bvAD) as proposed in the current work and the dysexecutive variant of AD as proposed elsewhere[85] in terms of behavioral features, cognitive performance and confirmation of AD pathology.

© 2021 Ossenkoppele R et al. *JAMA Neurology*

eREFERENCES:

1. Balasa M, Gelpi E, Antonell A, et al. Clinical features and APOE genotype of pathologically proven early-onset Alzheimer disease. *Neurology.* 2011;76(20):1720-1725.
2. Blennerhassett R, Lillo P, Halliday GM, Hodges JR, Kril JJ. Distribution of pathology in frontal variant Alzheimer's disease. *Journal of Alzheimer's disease : JAD.* 2014;39(1):63-70.
3. Phillips JS, Da Re F, Dratch L, et al. Neocortical origin and progression of gray matter atrophy in nonamnestic Alzheimer's disease. *Neurobiol Aging.* 2018;63:75-87.
4. Singleton E, Hansson O, Pijnenburg YAL, et al. Heterogeneous distribution of tau pathology in the behavioural variant of Alzheimer's disease. *Journal of Neurology, Neurosurgery & Psychiatry.* 2021:jnnp-2020-325497.
5. Woodward M, Jacova C, Black SE, et al. Differentiating the frontal variant of Alzheimer's disease. *International Journal of Geriatric Psychiatry.* 2010;25(7):732-738.
6. de Souza LC, Bertoux M, Funkiewiez A, et al. Frontal presentation of Alzheimer's disease: a series of patients with biological evidence by CSF biomarkers. *Dementia & neuropsychologia.* 2013;7(1):66-74.
7. Mendez MF, Joshi A, Tassniyom K, Teng E, Shapira JS. Clinicopathologic differences among patients with behavioral variant frontotemporal dementia. *Neurology.* 2013;80(6):561-568.
8. Fernandez-Calvo B, Ramos F, de Lucena VM. Frontal variant of Alzheimer's disease and typical Alzheimer's disease: A comparative study. *Anales De Psicologia.* 2013;29(1):293-300.
9. Ossenkoppele R, Pijnenburg YA, Perry DC, et al. The behavioural/dysexecutive variant of Alzheimer's disease: clinical, neuroimaging and pathological features. *Brain : a journal of neurology.* 2015;138(Pt 9):2732-2749.
10. Sala A, Caprioglio C, Santangelo R, et al. Brain metabolic signatures across the Alzheimer's disease spectrum. *Eur J Nucl Med Mol Imaging.* 2020;47(2):256-269.
11. Therriault J, Pascoal TA, Savard M, et al. Topographical distribution of amyloid-β, tau and atrophy in behavioral / dysexecutive AD patients. *Neurology.* 2020:10.1212/WNL.0000000000011081.
12. Bergeron D, Beauregard JM, Jean G, et al. Posterior Cingulate Cortex Hypometabolism in Non-Amnestic Variants of Alzheimer's Disease. *Journal of Alzheimers Disease.* 2020;77(4):1569-1577.
13. Lehingue E, Gueniat J, Jourdaa S, et al. Improving the Diagnosis of the Frontal Variant of Alzheimer's Disease with the DAPHNE Scale. *Journal of Alzheimers Disease.* 2021;79(4):1735-1745.
14. Brun A, Gustafson L. Distribution of cerebral degeneration in Alzheimer's disease. A clinico-pathological study. *Archiv fur Psychiatrie und Nervenkrankheiten.* 1976;223(1):15-33.
15. Shibayama H, Kitoh J. Electron microscopic structure of the Alzheimer's neurofibrillary changes in case of atypical senile dementia. *Acta Neuropathol.* 1978;41(3):229-234.
16. Shuttleworth EC. Atypical presentations of dementia of the Alzheimer type. *Journal of the American Geriatrics Society.* 1984;32(7):485-490.
17. Brun A. Frontal lobe degeneration of non-Alzheimer type. I. Neuropathology. *Archives of gerontology and geriatrics.* 1987;6(3):193-208.
18. Perani D, Di Piero V, Vallar G, et al. Technetium-99m HM-PAO-SPECT study of regional cerebral perfusion in early Alzheimer's disease. *J Nucl Med.* 1988;29(9):1507-1514.
19. Bird TD, Sumi SM, Nemens EJ, et al. Phenotypic heterogeneity in familial alzheimer's disease: A study of 24 kindreds. *Annals of Neurology.* 1989;25(1):12-25.
20. Grady CL, Haxby JV, Schapiro MB, et al. Subgroups in dementia of the Alzheimer type identified using positron emission tomography. *The Journal of neuropsychiatry and clinical neurosciences.* 1990;2(4):373-384.
21. Molchan SE, Martinez RA, Lawlor BA, Grafman JH, Sunderland T. Reflections of the self: atypical misidentification and delusional syndromes in two patients with Alzheimer's disease. *The British journal of psychiatry : the journal of mental science.* 1990;157:605-608.

© 2021 Ossenkoppele R et al. *JAMA Neurology*

22. Raux G, Gantier R, Thomas-Anterion C, et al. Dementia with prominent frontotemporal features associated with L113P presenilin 1 mutation. *Neurology.* 2000;55(10):1577-1578.
23. Rippon GA, Crook R, Baker M, et al. Presenilin 1 mutation in an african american family presenting with atypical Alzheimer dementia. *Archives of neurology.* 2003;60(6):884-888.
24. Yokota O, Terada S, Ishizu H, et al. Variability and heterogeneity in Alzheimer's disease with cotton wool plaques: a clinicopathological study of four autopsy cases. *Acta Neuropathol.* 2003;106(4):348-356.
25. Doran M, Larner AJ. Prominent behavioural and psychiatric symptoms in early-onset Alzheimer's disease in a sib pair with the presenilin-1 gene R269G mutation. *European archives of psychiatry and clinical neuroscience.* 2004;254(3):187-189.
26. Kertesz A, McMonagle P, Blair M, Davidson W, Munoz DG. The evolution and pathology of frontotemporal dementia. *Brain.* 2005;128(Pt 9):1996-2005.
27. Shi J, Shaw CL, Du Plessis D, et al. Histopathological changes underlying frontotemporal lobar degeneration with clinicopathological correlation. *Acta Neuropathol.* 2005;110(5):501-512.
28. Forman MS, Farmer J, Johnson JK, et al. Frontotemporal dementia: Clinicopathological correlations. *Annals of Neurology.* 2006;59(6):952-962.
29. Larner AJ. "Frontal variant Alzheimer's disease": a reappraisal. *Clinical neurology and neurosurgery.* 2006;108(7):705-708.
30. Alladi S, Xuereb J, Bak T, et al. Focal cortical presentations of Alzheimer's disease. *Brain.* 2007;130(Pt 10):2636-2645.
31. Rabinovici GD, Furst AJ, Neil JP, et al. 11C-PIB PET imaging in Alzheimer disease and frontotemporal lobar degeneration. *Neurology.* 2007;68(15):1205.
32. Snowden JS, Stopford CL, Julien CL, et al. Cognitive phenotypes in Alzheimer's disease and genetic risk. *Cortex; a journal devoted to the study of the nervous system and behavior.* 2007;43(7):835-845.
33. Taylor KI, Probst A, Miserez AR, Monsch AU, Tolnay M. Clinical course of neuropathologically confirmed frontal-variant Alzheimer's disease. *Nature clinical practice Neurology.* 2008;4(4):226-232.
34. Kile SJ, Ellis WG, Olichney JM, Farias S, DeCarli C. Alzheimer abnormalities of the amygdala with Klüver-Bucy syndrome symptoms: an amygdaloid variant of Alzheimer disease. *Archives of neurology.* 2009;66(1):125-129.
35. Bigio EH, Mishra M, Hatanpaa KJ, et al. TDP-43 pathology in primary progressive aphasia and frontotemporal dementia with pathologic Alzheimer disease. *Acta Neuropathol.* 2010;120(1):43-54.
36. Habek M, Hajnsek S, Zarkovic K, Chudy D, Mubrin Z. Frontal Variant of Alzheimer's Disease: Clinico-CSF-Pathological Correlation. *Canadian Journal of Neurological Sciences.* 2010;37(1):118-120.
37. Lehmann M, Rohrer JD, Clarkson MJ, et al. Reduced cortical thickness in the posterior cingulate gyrus is characteristic of both typical and atypical Alzheimer's disease. *Journal of Alzheimer's disease : JAD.* 2010;20(2):587-598.
38. Piscopo P, Talarico G, Crestini A, et al. A novel mutation in the predicted TMIII domain of the PSEN2 gene in an Italian pedigree with atypical Alzheimer's disease. *Journal of Alzheimer's disease : JAD.* 2010;20(1):43-47.
39. Rabinovici GD, Rosen HJ, Alkalay A, et al. Amyloid vs FDG-PET in the differential diagnosis of AD and FTLD. *Neurology.* 2011;77(23):2034-2042.
40. Snowden JS, Thompson JC, Stopford CL, et al. The clinical diagnosis of early-onset dementias: diagnostic accuracy and clinicopathological relationships. *Brain.* 2011;134(Pt 9):2478-2492.
41. Whitwell JL, Jack CR, Przybelski SA, et al. Temporoparietal atrophy: A marker of AD pathology independent of clinical diagnosis. *Neurobiology of Aging.* 2011;32(9):1531-1541.
42. Borroni B, Pilotto A, Bonvicini C, et al. Atypical presentation of a novel Presenilin 1 R377W mutation: sporadic, late-onset Alzheimer disease with epilepsy and frontotemporal atrophy.

© 2021 Ossenkoppele R et al. *JAMA Neurology*

Neurological sciences : official journal of the Italian Neurological Society and of the Italian Society of Clinical Neurophysiology. 2012;33(2):375-378.

43. Duker AP, Espay AJ, Wszolek ZK, Rademakers R, Dickson DW, Kelley BJ. Atypical motor and behavioral presentations of Alzheimer disease: a case-based approach. *The neurologist.* 2012;18(5):266-272.
44. Wallon D, Rousseau S, Rovelet-Lecrux A, et al. The French series of autosomal dominant early onset Alzheimer's disease cases: mutation spectrum and cerebrospinal fluid biomarkers. *Journal of Alzheimer's disease : JAD.* 2012;30(4):847-856.
45. Herrero-San Martin A, Villarejo-Galende A, Rabano-Gutierrez A, Guerrero-Marquez C, Porta-Etessam J, Bermejo-Pareja F. Frontal variant of Alzheimer's disease. Two pathologically confirmed cases and a literature review. *Revista De Neurologia.* 2013;57(12):542-548.
46. Marini S, Lucidi G, Tedde A, Bessi V, Nacmias B. A case of atypical early-onset Alzheimer's disease carrying the missense mutation Thr354Ile in exon 10 of the PSEN1 gene. *Neurological sciences : official journal of the Italian Neurological Society and of the Italian Society of Clinical Neurophysiology.* 2013;34(9):1691-1692.
47. Léger GC, Banks SJ. Neuropsychiatric symptom profile differs based on pathology in patients with clinically diagnosed behavioral variant frontotemporal dementia. *Dementia and geriatric cognitive disorders.* 2014;37(1-2):104-112.
48. Nygaard HB, Lippa CF, Mehdi D, Baehring JM. A Novel Presenilin 1 Mutation in Early-Onset Alzheimer's Disease With Prominent Frontal Features. *American journal of Alzheimer's disease and other dementias.* 2014;29(5):433-435.
49. Balasa M, Gelpi E, Martín I, et al. Diagnostic accuracy of behavioral variant frontotemporal dementia consortium criteria (FTDC) in a clinicopathological cohort. *Neuropathology and applied neurobiology.* 2015;41(7):882-892.
50. Paterson RW, Toombs J, Slattery CF, et al. Dissecting IWG-2 typical and atypical Alzheimer's disease: insights from cerebrospinal fluid analysis. *J Neurol.* 2015;262(12):2722-2730.
51. Woodward MC, Rowe CC, Jones G, Villemagne VL, Varos TA. Differentiating the frontal presentation of Alzheimer's disease with FDG-PET. *Journal of Alzheimer's disease : JAD.* 2015;44(1):233-242.
52. Li P, Zhou YY, Lu D, Wang Y, Zhang HH. Correlated patterns of neuropsychological and behavioral symptoms in frontal variant of Alzheimer disease and behavioral variant frontotemporal dementia: a comparative case study. *Neurological sciences : official journal of the Italian Neurological Society and of the Italian Society of Clinical Neurophysiology.* 2016;37(5):797-803.
53. Ossenkoppele R, Schonhaut DR, Scholl M, et al. Tau PET patterns mirror clinical and neuroanatomical variability in Alzheimer's disease. *Brain : a journal of neurology.* 2016;139(Pt 5):1551-1567.
54. Scialò C, Ferrara M, Accardo J, et al. Frontal Variant Alzheimer Disease or Frontotemporal Lobe Degeneration With Incidental Amyloidosis? *Alzheimer Dis Assoc Disord.* 2016;30(2):183-185.
55. Dickerson BC, McGinnis SM, Xia C, et al. Approach to atypical Alzheimer's disease and case studies of the major subtypes. *CNS spectrums.* 2017;22(6):439-449.
56. Duclos H, de La Sayette V, Bonnet AL, et al. Social Cognition in the Frontal Variant of Alzheimer's Disease: A Case Study. *Journal of Alzheimer's disease : JAD.* 2017;55(2):459-463.
57. Kawakatsu S, Kobayashi R, Hayashi H. Typical and atypical appearance of early-onset Alzheimer's disease: A clinical, neuroimaging and neuropathological study. *Neuropathology : official journal of the Japanese Society of Neuropathology.* 2017;37(2):150-173.
58. Oboudiyat C, Gefen T, Varelas E, et al. Cerebrospinal fluid markers detect Alzheimer's disease in nonamnestic dementia. *Alzheimers Dement.* 2017;13(5):598-601.
59. Perry DC, Brown JA, Possin KL, et al. Clinicopathological correlations in behavioural variant frontotemporal dementia. *Brain.* 2017;140(12):3329-3345.

© 2021 Ossenkoppele R et al. *JAMA Neurology*

60. Rawtaer I, Krishnamoorthy A. Co-occurring frontal variant Alzheimer's dementia and carrier of Huntington's disease allele with reduced penetrance. *Psychogeriatrics : the official journal of the Japanese Psychogeriatric Society.* 2017;17(6):488-490.
61. Sawyer RP, Rodriguez-Porcel F, Hagen M, Shatz R, Espay AJ. Diagnosing the frontal variant of Alzheimer's disease: a clinician's yellow brick road. *Journal of clinical movement disorders.* 2017;4:2.
62. Bagyinszky E, Lee HM, Van Giau V, et al. PSEN1 p.Thr116Ile Variant in Two Korean Families with Young Onset Alzheimer's Disease. *International journal of molecular sciences.* 2018;19(9).
63. Boon BDC, Hoozemans JJM, Lopuhaä B, et al. Neuroinflammation is increased in the parietal cortex of atypical Alzheimer's disease. *Journal of Neuroinflammation.* 2018;15(1):170.
64. Seo SW, Thibodeau M-P, Perry DC, et al. Early vs late age at onset frontotemporal dementia and frontotemporal lobar degeneration. *Neurology.* 2018;90(12):e1047-e1056.
65. Whitwell JL, Graff-Radford J, Tosakulwong N, et al. Imaging correlations of tau, amyloid, metabolism, and atrophy in typical and atypical Alzheimer's disease. *Alzheimers Dement.* 2018;14(8):1005-1014.
66. de Souza LC, Mariano LI, de Moraes RF, Caramelli P. Behavioral variant of frontotemporal dementia or frontal variant of Alzheimer's disease? A case study. *Dementia & neuropsychologia.* 2019;13(3):356-360.
67. Foiani MS, Cicognola C, Ermann N, et al. Searching for novel cerebrospinal fluid biomarkers of tau pathology in frontotemporal dementia: an elusive quest. *J Neurol Neurosurg Psychiatry.* 2019;90(7):740-746.
68. Monacelli F, Martella L, Parodi MN, Odetti P, Fanelli F, Tabaton M. Frontal Variant of Alzheimer's Disease: A Report of a Novel PSEN1 Mutation. *Journal of Alzheimer's disease : JAD.* 2019;70(1):11-15.
69. Nolan A, Resende EDF, Petersen C, et al. Astrocytic Tau Deposition Is Frequent in Typical and Atypical Alzheimer Disease Presentations. *Journal of Neuropathology and Experimental Neurology.* 2019;78(12):1112-1123.
70. Pawlowski M, Joksch V, Wiendl H, Meuth SG, Duning T, Johnen A. Apraxia screening predicts Alzheimer pathology in frontotemporal dementia. *J Neurol Neurosurg Psychiatry.* 2019;90(5):562-569.
71. Phillips JS, Da Re F, Irwin DJ, et al. Longitudinal progression of grey matter atrophy in non-amnestic Alzheimer's disease. *Brain.* 2019;142(6):1701-1722.
72. Pillai JA, Bonner-Jackson A, Bekris LM, Safar J, Bena J, Leverenz JB. Highly Elevated Cerebrospinal Fluid Total Tau Level Reflects Higher Likelihood of Non-Amnestic Subtype of Alzheimer's Disease. *Journal of Alzheimer's disease : JAD.* 2019;70(4):1051-1058.
73. Tan RH, Yang Y, McCann H, Shepherd C, Halliday GM. Von Economo Neurons in Behavioral Variant Frontotemporal Dementia with Underlying Alzheimer's Disease. *Journal of Alzheimer's disease : JAD.* 2019;69(4):963-967.
74. Wang Y, Shi Z, Zhang N, et al. Spatial Patterns of Hypometabolism and Amyloid Deposition in Variants of Alzheimer's Disease Corresponding to Brain Networks: a Prospective Cohort Study. *Molecular imaging and biology.* 2019;21(1):140-148.
75. Wong S, Strudwick J, Devenney E, Hodges JR, Piguet O, Kumfor F. Frontal variant of Alzheimer's disease masquerading as behavioural-variant frontotemporal dementia: a case study comparison. *Neurocase.* 2019;25(1-2):48-58.
76. Cai H, Ning S, Li W, Li X, Xiao S, Sun L. Patient with frontal-variant syndrome in early-onset Alzheimer's disease. *General psychiatry.* 2020;33(2):e100173.
77. Cousins KAQ, Irwin DJ, Wolk DA, et al. ATN status in amnestic and non-amnestic Alzheimer's disease and frontotemporal lobar degeneration. *Brain.* 2020;143(7):2295-2311.
78. Li CH, Fan SP, Chen TF, Chiu MJ, Yen RF, Lin CH. Frontal variant of Alzheimer's disease with asymmetric presentation mimicking frontotemporal dementia: Case report and literature review. *Brain and behavior.* 2020;10(3):e01548.

© 2021 Ossenkoppele R et al. *JAMA Neurology*

79. Paquin V, Therriault J, Pascoal TA, Rosa-Neto P, Gauthier S. Frontal Variant of Alzheimer Disease Differentiated From Frontotemporal Dementia Using in Vivo Amyloid and Tau Imaging. *Cognitive and behavioral neurology : official journal of the Society for Behavioral and Cognitive Neurology.* 2020;33(4):288-293.
80. Scarioni M, Gami-Patel P, Timar Y, et al. Frontotemporal Dementia: Correlations Between Psychiatric Symptoms and Pathology. *Annals of Neurology.* 2020;87(6):950-961.
81. Singleton EH, Pijnenburg YAL, Sudre CH, et al. Investigating the clinico-anatomical dissociation in the behavioral variant of Alzheimer disease. *Alzheimers Res Ther.* 2020;12(1):148.
82. Bergeron D, Sellami L, Poulin S, Verret L, Bouchard RW, Laforce R. The Behavioral/Dysexecutive Variant of Alzheimer's Disease: A Case Series with Clinical, Neuropsychological, and FDG-PET Characterization. *Dementia and Geriatric Cognitive Disorders.* 2021;49(5):518-525.
83. Zhu L, Sun LM, Sun L, Xiao SF. Case of early-onset Alzheimer's disease with atypical manifestation. *General Psychiatry.* 2021;34(1).
84. Eikelboom WS, van den Berg E, Singleton EH, et al. Neuropsychiatric and Cognitive Symptoms Across the Alzheimer Disease Clinical Spectrum: Cross-sectional and Longitudinal Associations. *Neurology.* 2021.
85. Townley RA, Graff-Radford J, Mantyh WG, et al. Progressive dysexecutive syndrome due to Alzheimer's disease: a description of 55 cases and comparison to other phenotypes. *Brain Commun.* 2020;2(1):fcaa068.

© 2021 Ossenkoppele R et al. *JAMA Neurology*
